# Astaxanthin protects against early acute kidney injury in severely burned rats by inactivating the TLR4/MyD88/NF-κB axis and upregulating heme oxygenase-1

**DOI:** 10.1038/s41598-021-86146-w

**Published:** 2021-03-23

**Authors:** Songxue Guo, Linsen Guo, Quan Fang, Meirong Yu, Liping Zhang, Chuangang You, Xingang Wang, Yong Liu, Chunmao Han

**Affiliations:** 1 Department of Plastic Surgery, The Second Affiliated Hospital Zhejiang University School of Medicine, 1511 Jianghong Road, Hangzhou, 310000 Zhejiang China; 2 Department of Burns, Changzhou No.7 People’s Hospital, 288 East Yanling Road, Changzhou, 213011 Jiangsu China; 3 Clinical Research Center, The Second Affiliated Hospital Zhejiang University School of Medicine, 88 Jiefang Road, Hangzhou, 310009 Zhejiang China; 4 Department of Burns, The Second Affiliated Hospital Zhejiang University School of Medicine, 88 Jiefang Road, Hangzhou, 310009 Zhejiang China; 5 West China Hospital, Sichuan University, 37 Guoxuexiang Street, Chengdu, China

**Keywords:** Acute kidney injury, Trauma

## Abstract

Early acute kidney injury (AKI) contributes to severe morbidity and mortality in critically burned patients. Renal inflammation plays a vital role in the progression of early AKI, acting as a therapeutic target. Astaxanthin (ATX) is a strong antioxidant widely distributed in marine organisms that exerts many biological effects in trauma and disease. ATX is also suggested to have anti-inflammatory activity. Hence, we attempted to explore the role of ATX in protecting against early postburn AKI via its anti-inflammatory effects and the related mechanisms. A severely burned model was established for histological and biochemical assessments based on adult male rats. We found that oxidative stress-induced tissue inflammation participated in the development of early AKI after burn injury and that the MyD88-dependent TLR4/NF-κB pathway was activated to regulate renal inflammation. The TLR4 and NF-κB inhibitors TAK242 and PDTC showed similar effects in attenuating burn-induced renal inflammation and early AKI. Upon ATX treatment, the release of inflammatory mediators in the kidneys was downregulated, while the TLR4/MyD88/NF-κB axis was inhibited in a dose-related manner. TAK242 and PDTC could enhance the anti-inflammatory effect of high-dose ATX, whereas lipopolysaccharide (LPS) reversed its action. Furthermore, the expression of heme oxygenase (HO)-1 was upregulated by ATX in a dose-related manner. Collectively, the above data suggest that ATX protects against renal inflammation in a dose-related manner by regulating the TLR4/MyD88/NF-κB axis and HO-1 and ultimately prevents early AKI following severe burns.

## Introduction

Severely burned patients (total body surface area (TBSA) ≥ 20%) must face a series of devastating circulatory or organ issues after the initial thermal insult, and these issues can largely be attributed to burn-induced ischemia/hyporeperfusion, oxidative damage, and secondary inflammation or cell death^1–3^. As an organ with a rich blood supply and polyunsaturated fatty acids, the kidney is vulnerable to attack from reactive oxygen species (ROS) and the subsequent inflammatory response under ischemic conditions, leading to development of AKI^4,5^. In terms of burn injury, early acute kidney injury (AKI) usually occurs within the first 3 days after trauma, contributes to a poor prognosis and even high mortality (80%) and morbidity, is apt to give rise to a series of rapid pathological changes in tubular structures and predisposes patients to sequential functional deterioration of the kidney^2,6^. The incidence of acute renal dysfunction in burn patients receiving critical care can be up to 40%^2^. We previously provided several lines of evidence to support the key roles of ROS-related oxidative stress and secondary local mitochondria-related apoptosis in pathophysiological issues during the progression of burn-induced early AKI^7^. Furthermore, we found clues indicating that renal tissue inflammation should be taken into account as another important issue following ischemia or ROS burst^8^. Multiple studies investigating severe trauma (including burns), large surgery and sepsis have suggested the effectiveness of anti-inflammatory strategies to protect against AKI or other acute organ injuries following the above conditions^8–10^. However, early and timely interventions for burn-induced organ inflammation are still worthy of further exploration.

Nuclear factor (NF)-κB is regarded as a potential and crucial intermediate regulator of local inflammatory conditions in early AKI after burn injury^8^. We observed that the selective antioxidant molecular hydrogen may inhibit burn-induced release of inflammatory cytokines in rat kidneys through an NF-κB-mediated signaling pathway and ultimately attenuate early AKI and reduce apoptosis^8^. Furthermore, the Toll-like receptor (TLR)4/NF-κB signaling pathway has been suggested to be a classic cascade that is able to regulate development of the inflammatory response in diverse organ injuries following ischemia–reperfusion (IR)^11^. Moreover, the effects of nephroprotectants and preconditioning treatment on different types of kidney injury are closely related to downregulation of signals included in the TLR4/NF-κB pathway^5,12^. Recently, several researchers have observed a regulatory role of the TLR4 pathway in burn-induced sepsis and lung or cerebral injury, indicating that it is a promising therapeutic target. In addition to inflammation, a reactive oxygen species (ROS) burst is also accompanied by the expression of endogenous antioxidant proteins, such as heme oxygenase-1 (HO-1) and Nrf2, in some ischemic or LPS-induced organ injuries as a self-defense response^13,14^. HO-1 has been reported to have a nephroprotective role in renal injury caused by diverse stimuli due to its antagonism against oxidative damage and its regulation of several biological processes^13,15,16^. Considering burn-induced hypodynamic circulation and increased oxidative stress, the TLR4/NF-κB pathway and HO-1 are potential players in the development of burn-induced early AKI.

Astaxanthin (3,3′-dihydroxy-b,b'-carotene-4,4′-dione, ATX) is a natural carotenoid easily obtained from marine organisms and exhibits more robust and powerful antioxidative effects than other carotenoids^17^. Previously, researchers demonstrated that pretreatment or immediate administration of ATX can attenuate oxidative stress-induced toxicity in tubular epithelial cells and I/R-induced or diabetes-related renal injury in mice by reducing oxidative stress, inflammation and tubular apoptosis^18,19^. In terms of burn injury, we observed that ATX exerted a protective effect against burn-induced early AKI by ameliorating oxidative damage and downregulating mitochondria-related apoptosis via the PI3K/Akt/Bad pathway^7^. Combined with similar reports of its effects on oxidative damage and inflammation in other organ injuries, ATX seems to be a promising and nontoxic therapeutic reagent to prevent early AKI after burn injury^20,21^.

Given the important roles of oxidative stress and secondary renal inflammation in severe burn-induced early AKI, we hypothesized a possible role of ATX in protection via its anti-inflammatory effects and the potential mechanisms of action in early postburn AKI through the regulation of the TLR4/NF-κB pathway and HO-1, we and aimed to explore more details to support clinical treatment.

## Results

### Severe burn induced acute kidney injury in the early stage after injury, accompanied by increased oxidative stress and renal inflammation, whereas TAK242 or PDTC attenuated most of the above changes

**Figure 1:**
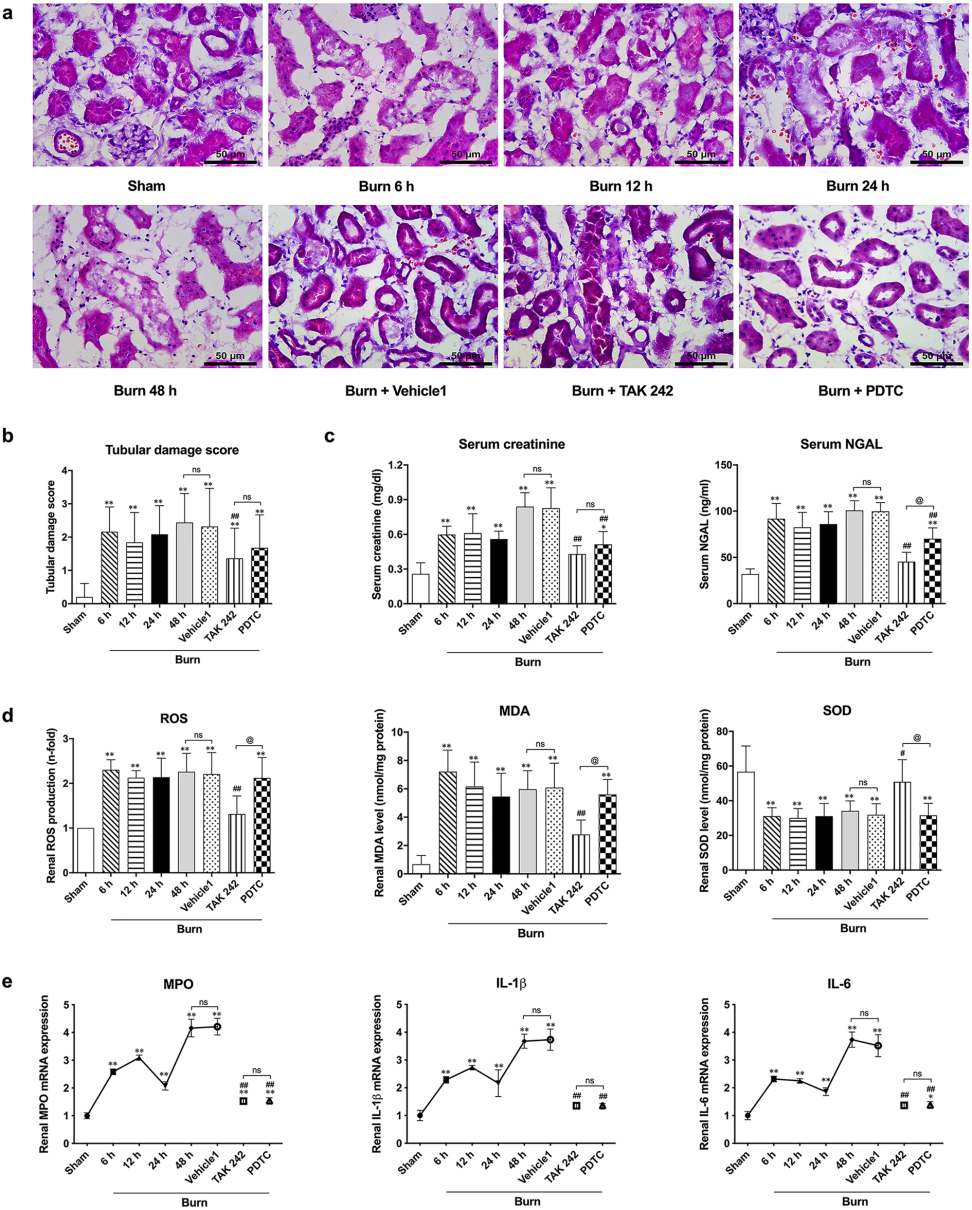
Histological, functional and biological assessments of kidney after burn. (**a**) Representative HE-stained images of renal tissues at a magnification of 200 × ; (**b**) Tubular damage score according to HE-stained slices; (**c**) Blood-based detection of creatinine and NGAL; (**d**) Oxidative stress evaluation based on commercial kits of ROS, MDA and SOD; (**e**) The release levels of inflammatory mediators after insults. n = 8 for each group. The results are expressed as the mean ± SD. ^*^p < 0.05, ^**^p < 0.01, v.s. sham; ^#^*p* < 0.05, ^##^*p* < 0.01, v.s. vehicle1; ^@^*p* < 0.05, ^ns^*p* > 0.05.

Similar to a previous report, under a microscope, hematoxylin and eosin (HE)-stained sections showed typical histological changes in the kidney after burn injury, mainly including the absence of the proximal tubular brush border, blebbing of apical membranes, separation of tubular epithelial cells from the basement membrane, or aggregation of cells and proteins in the luminal region (Fig. 1a). Interestingly, unlike vehicle 1, application of both the selective TLR4 inhibitor TAK242 and the NF-κB activation inhibitor PDTC seemed to attenuate burn-induced histological damage in the kidneys (Fig. 1a). Furthermore, the tubular damage scores showed a gradual and significant increase from 6 h until 48 h post burn injury (Fig. 1b). The scores of the TAK242 and PDTC groups were significantly lower than those of the 48 h group after burn injury, while there was no difference between the scores of the vehicle 1 and burn groups at 48 h post burn injury (Fig. 1b).

In terms of renal function, similarly, we observed that burn injury increased the levels of sCr and NGAL at 6 h post burn injury, and levels peaked at 48 h after burn injury, whereas TAK242 had a stronger effect on attenuating the burn-induced increase in sCr and NGAL than PDTC. Vehicle1 had no effect on the levels of sCr and NGAL in rats at 48 h after burn injury (Fig. 1c).

In terms of oxidative status and secondary inflammation, we observed an obvious ROS burst in rat kidneys beginning in the early stage (6 h) after burn injury that persisted until 48 h post burn injury (Fig. 1d). Similarly, renal MDA levels exhibited a significant increase from 6 to 48 h after burn injury (Fig. 1d). Conversely, the representative endogenous antioxidant enzyme SOD showed a marked decrease after burn injury (Fig. 1d). The increased renal oxidative stress after burn injury conformed to the results of our previous study; nevertheless, neither vehicle 1 nor PDTC affected the burn-induced changes in ROS generation or the levels of MDA and SOD (Fig. 1d). However, interestingly, TAK242 significantly decreased the ROS burst and the MDA level in burned rat kidneys 48 h after burn injury (Fig. 1d). Furthermore, the renal mRNA levels of typical inflammatory mediators, such as MPO, IL-1β and IL-6, in burned rats increased significantly and gradually after insult and peaked 48 h after burn injury, although a slight decrease occurred 24 h post burn injury compared to the obvious elevation observed 6 h and 12 h post burn injury (Fig. 1e). Vehicles showed no effect on the mRNA transcription of MPO, IL-1β and IL-6, while both TAK242 and PDTC markedly lowered the burn-induced increase in MPO, IL-1β and IL-6 48 h after burn insult (Fig. 1e).

### Activation of the TLR4/MyD88/NF-κB signaling pathway contributed to regulation of renal inflammation following burn injury

**Figure 2:**
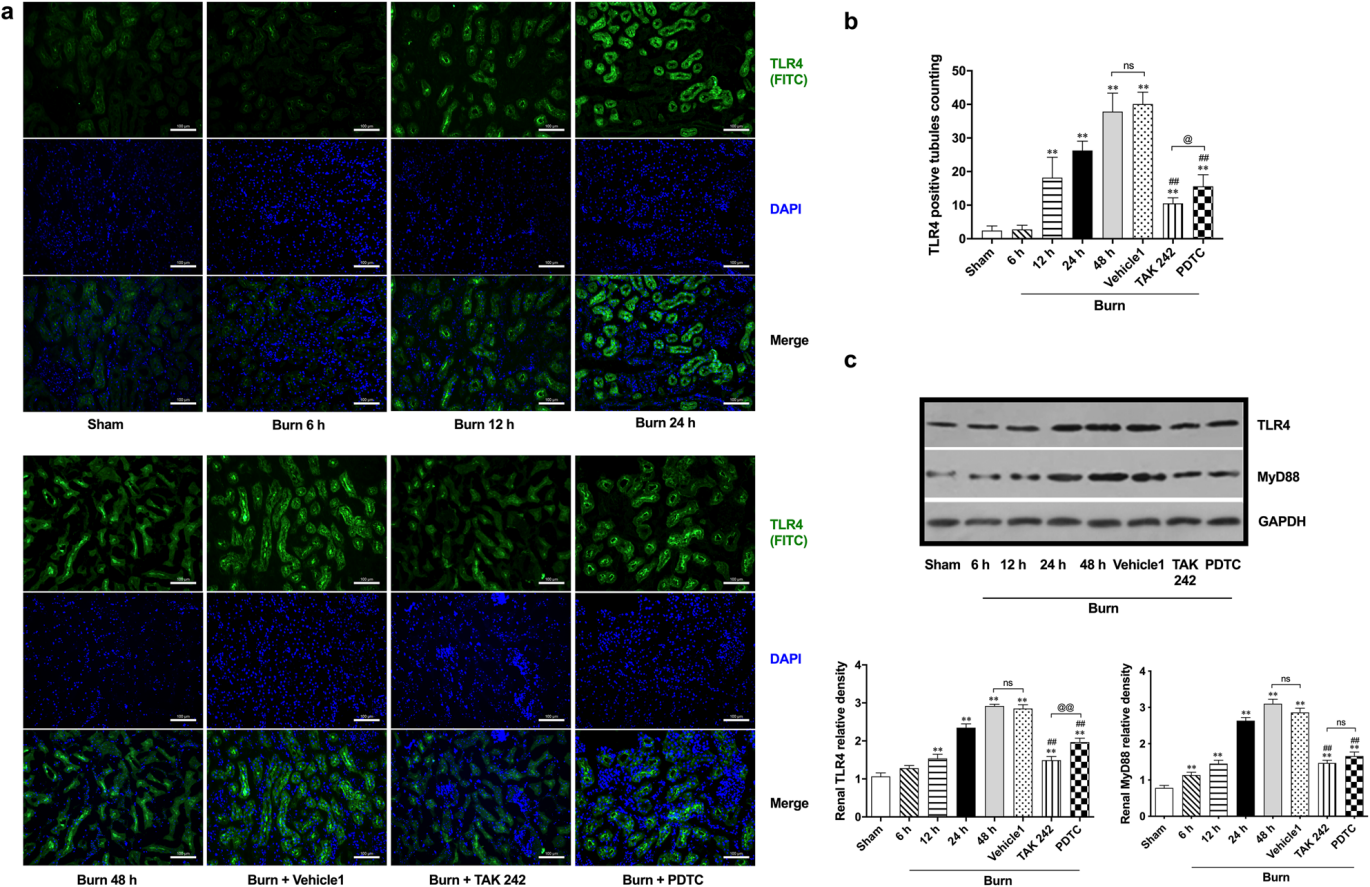
Immunofluorescent and immunoblotting detection of TLR4 pathway. (**a**) Immunofluorescence double staining of TLR4; (**b**) The double-blind counting of TLR4 positively-stained tubules (green-labeled), n = 12 per group; (**c**) Representative bands of Western blot on TLR4 and MyD88, n = 6 for each group. The results are expressed as the mean ± SD. ^*^p < 0.05, ^**^p < 0.01, v.s. sham; ^#^*p* < 0.05, ^##^*p* < 0.01, v.s. vehicle1; ^@@^*p* < 0.01, ^ns^*p* > 0.05.

We also investigated potential signaling pathways related to renal inflammation induced by burn injury. According to the immunofluorescence results and subsequent tubule count, typical TLR4-positive (FITC-labeled, green) renal tubules appeared 12 h post burn injury, representing an increased distribution of TLR4 (Fig. 2a,b). The increases continued to 48 h post burn (Fig. 2a and b). In contrast to the slight effect of vehicle, both TAK242 and PDTC were able to reduce the burn-induced increase in TLR4-positive tubules 48 h after insult, and the effect of TAK242 seemed to be more obvious (Fig. 2a,b). Further immunoblot detection supported the observation based on immunofluorescence staining, and the expression of TLR4 showed a similar tendency (Fig. 2c). Specifically, TLR4 expression was significantly upregulated from 6 h post burn injury until 48 h, while there was only a slight increase at 6 h after burn injury. TAK242 exhibited a stronger effect in reversing the marked increase in TLR4 expression than PDTC. Vehicle exerted no regulation on TLR4 expression. In view of the potential importance of MyD88 in the TLR4 signaling pathway, we investigated the expression of MyD88 in rat kidneys after burn injury (Fig. 2c). The results showed that the expression of MyD88 significantly increased beginning at 6 h post burn injury and showed a gradual increasing tendency over time, similar to TLR4. TAK242 and PDTC obviously downregulated MyD88 expression 48 h post burn injury, while there were no differences between the 48 h burn and vehicle groups.

**Figure 3:**
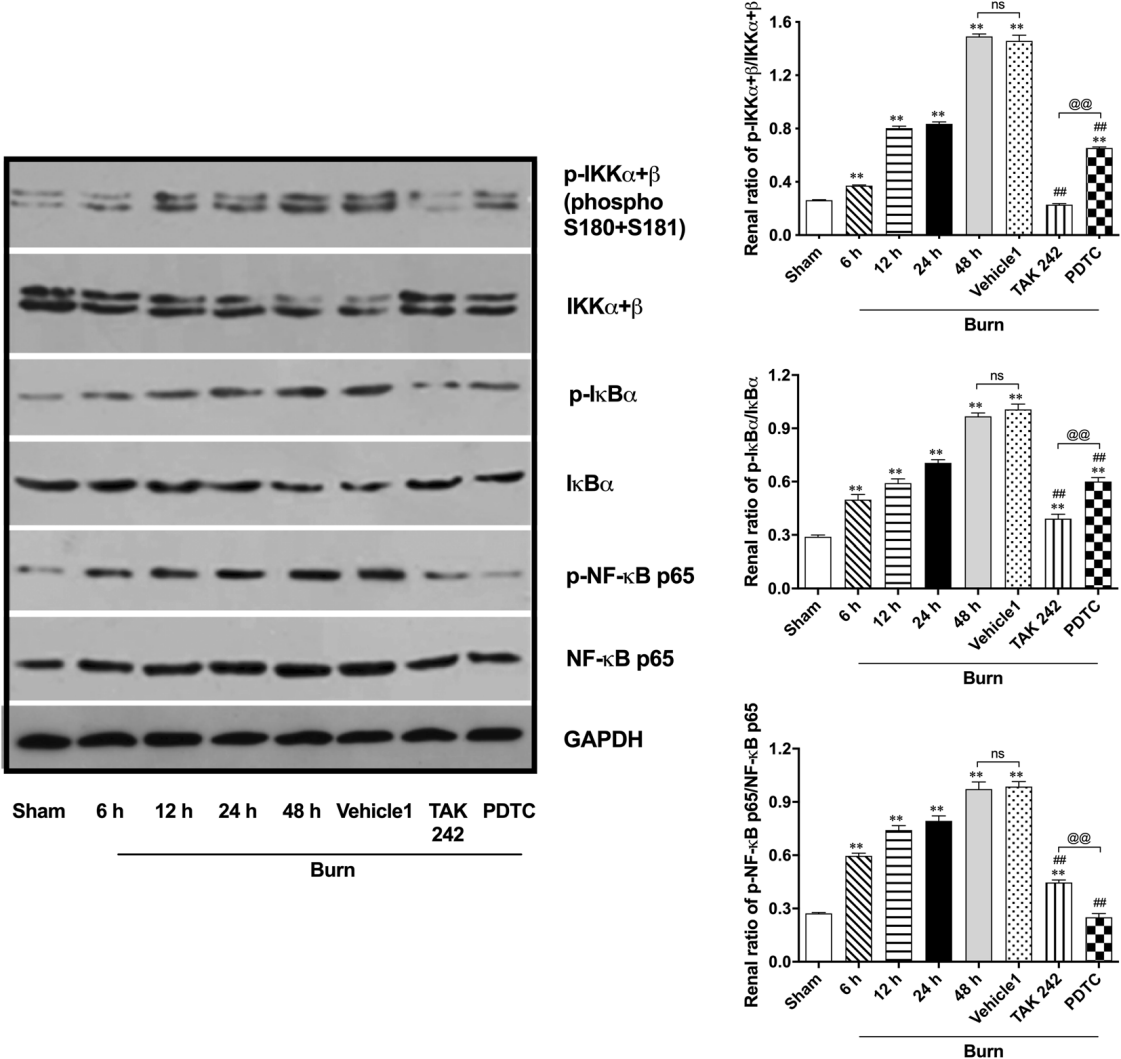
Immunoblotting detection on downstream signals of MyD88 dependent TLR4 pathway. n = 6 for each group. The results are expressed as the mean ± SD. ^*^p < 0.05, ^**^p < 0.01, v.s. sham; ^#^*p* < 0.05, ^##^*p* < 0.01, v.s. vehicle1; ^@^*p* < 0.05, ^@@^*p* < 0.01, ^ns^*p* > 0.05.

Further immunoblot detection was performed to determine the possible activation of signals downstream of TLR4/MyD88 (Fig. 3). Finally, we observed increased phosphorylation of inhibitor of nuclear factor kappa-B kinase subunit (IKK)α + β, NF-kappa-B inhibitor (IκB)α, and NF-κB p65 beginning at 6 h after burn injury. All three peaks appeared at 48 h. The inhibitory effect of TAK242 on the burn–induced phosphorylation of IKKα + β and IκBα in kidneys was significant, whereas PDTC showed a more powerful ability to downregulate NF-κB p65 phosphorylation than TAK242. However, both were able to reverse the burn-induced phosphorylation of IKKα + β, IκBα, and NF-κB p65 at 48 h after insult, while vehicle showed no effect on phosphorylation of these proteins.

All the above results indicate that typical activation of the MyD88-dependent TLR4/NF-κB signaling pathway might be involved in the induction of early renal inflammation after burn injury.

### ATX dose-relatedly attenuated burn-induced tissue inflammation in rat kidneys

**Figure 4:**
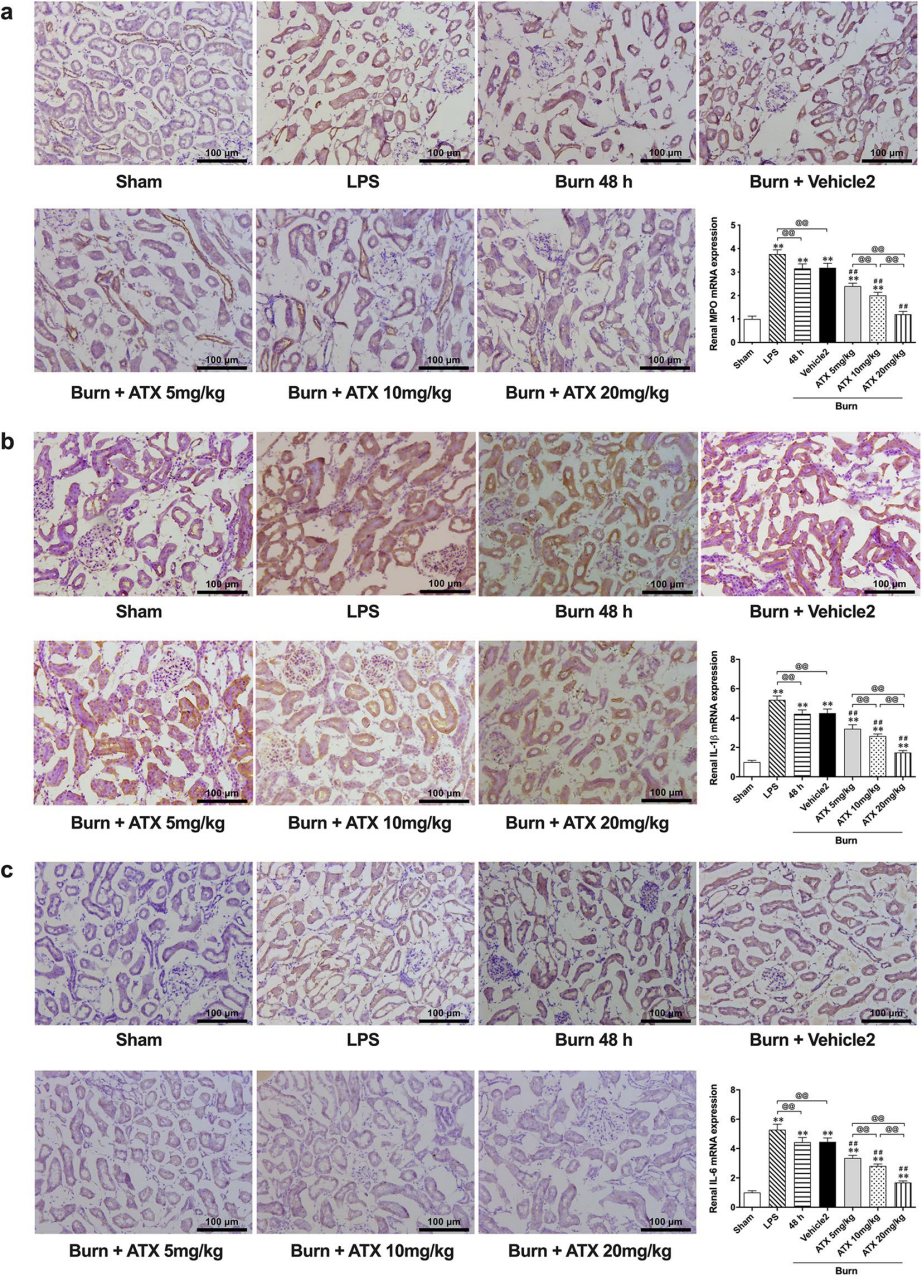
Astaxanthin dose-relatively decreases the burn-induced release of inflammatory mediators. (**a**) Representative immunohistochemical images and tissue mRNA levels of MPO with ATX treatment; (**b**) Representative immunohistochemical images and tissue mRNA levels of IL-1β with ATX treatment; (**c**) Representative immunohistochemical images and tissue mRNA levels of IL-6 with ATX treatment. n = 6 for each group. The results are expressed as the mean ± SD. ^*^p < 0.05, ^**^p < 0.01, v.s. sham; ^#^*p* < 0.05, ^##^*p* < 0.01, v.s. vehicle2; ^@^*p* < 0.05, ^@@^*p* < 0.01, ^ns^*p* > 0.05.

In our previous study, we observed the typical distribution of inflammatory mediators in burned rat renal tissue based on the results of immunohistochemical (IHC) staining. In this study, according to the results of IHC staining, we detected increased MPO, IL-1β and IL-6 immunoreactivity in the renal tubules of burned rats at the selected time window, similar to the groups treated with the classic selective TLR4 activator lipopolysaccharide (LPS) or the ATX solvent (vehicle 2) (Fig. 4). However, ATX seemed to dose-relatedly decrease the number of MPO-, IL-1β- or IL-6-positive tubular cells in rats 48 h after burn injury (Fig. 4). The results of IHC assessment were further supported by qRT-PCR detection (Fig. 4). The burn 48 h and vehicle groups exhibited similar expression of different inflammatory mediators, such as MPO, IL-1β and IL-6, although both showed slightly weaker effects than the LPS group. The levels of MPO, IL-1β and IL-6 in all three groups were significantly higher than in the sham group. All doses of ATX downregulated burn-increased MPO, IL-1β and IL-6 mRNA expression at 48 h after burn injury and displayed a dose-related effect. The effect peaked at the dose of 20 mg/kg. All these results suggest that ATX might dose-relatedly attenuate burn-induced inflammation in rat kidneys.

### ATX downregulated activation of the TLR4/MyD88/NF-κB signaling pathway in kidneys after burn injury in a dose-related manner

**Figure 5:**
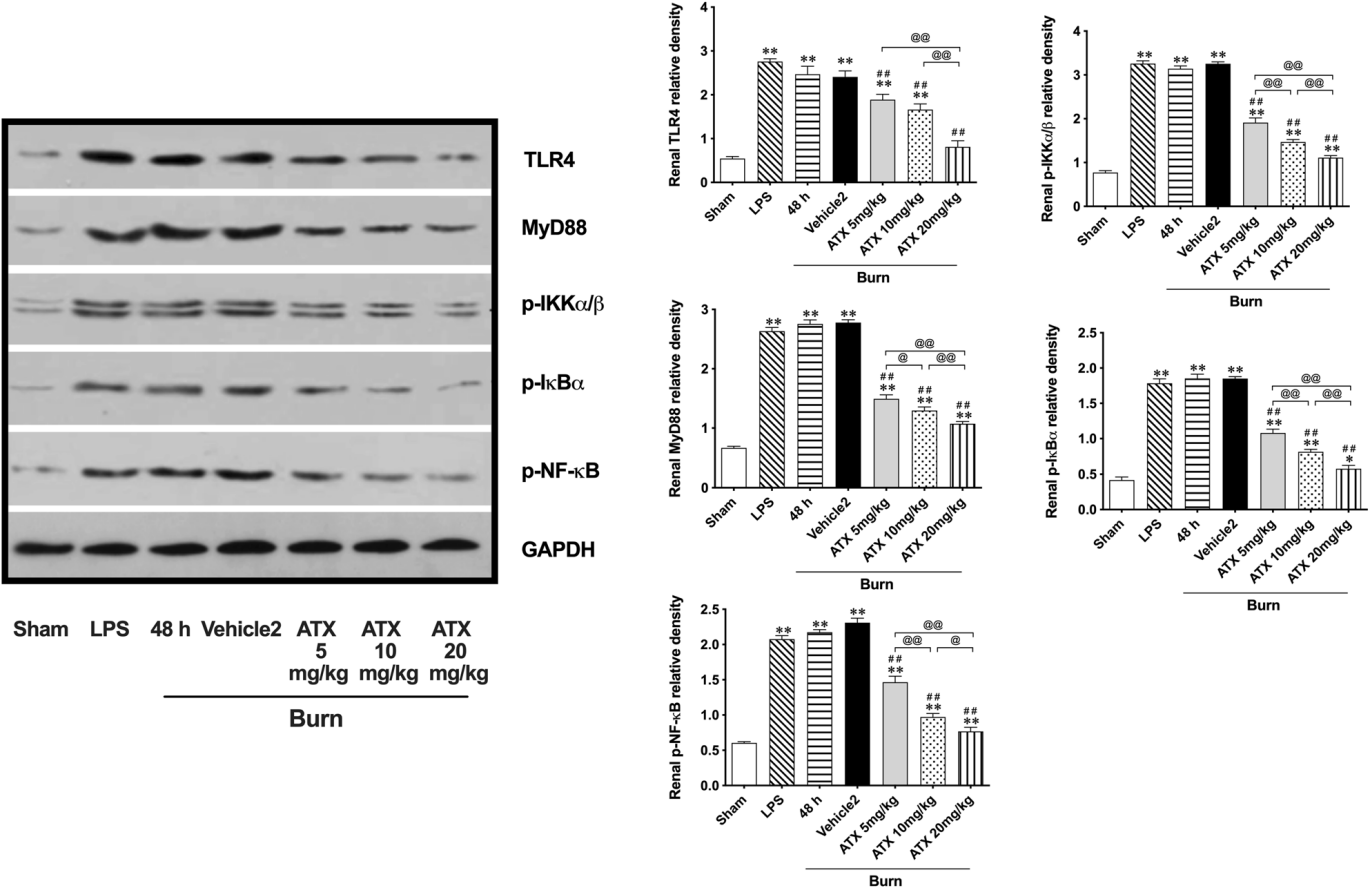
Astaxanthin dose-relatively downregulates the activation of TLR4/myD88/NF-κB signaling pathway. n = 6 for each group. The results are expressed as the mean ± SD. ^*^ p < 0.05, ^**^ p < 0.01, *v.s.* sham; ^#^ p < 0.05, ^##^ p < 0.01, *v.s.* vehicle2; ^@^ p < 0.05, ^@@^ p < 0.01, ^ns^ p > 0.05.

According to the aforementioned role of the TLR4/MyD88/NF-κB signaling pathway in regulating burn-induced renal tissue inflammation, we investigated the potential effect of ATX on the pathway (Fig. 5). Western blotting results showed that both LPS and burn insult induced significant elevation of TLR4 and MyD88 and secondary phosphorylation of IKKα + β, IκBα, and NF-κB p65 compared to the sham group. All three doses of ATX were able to remarkably downregulate TLR4 and MyD88 expression, and the strongest effect appeared in the 20 mg/kg ATX group. In addition, downstream downregulation of IKKα + β, IκBα, and NF-κB p65 phosphorylation was detected in all three ATX treatment groups. The peak of regulation was dose-dependent. Overall, ATX seemed to inhibit activation of the TLR4/MyD88/NF-κB signaling cascade.

### ATX ameliorated burn-induced renal inflammation through the MyD88-dependent TLR4/NF-κB pathway

**Figure 6:**
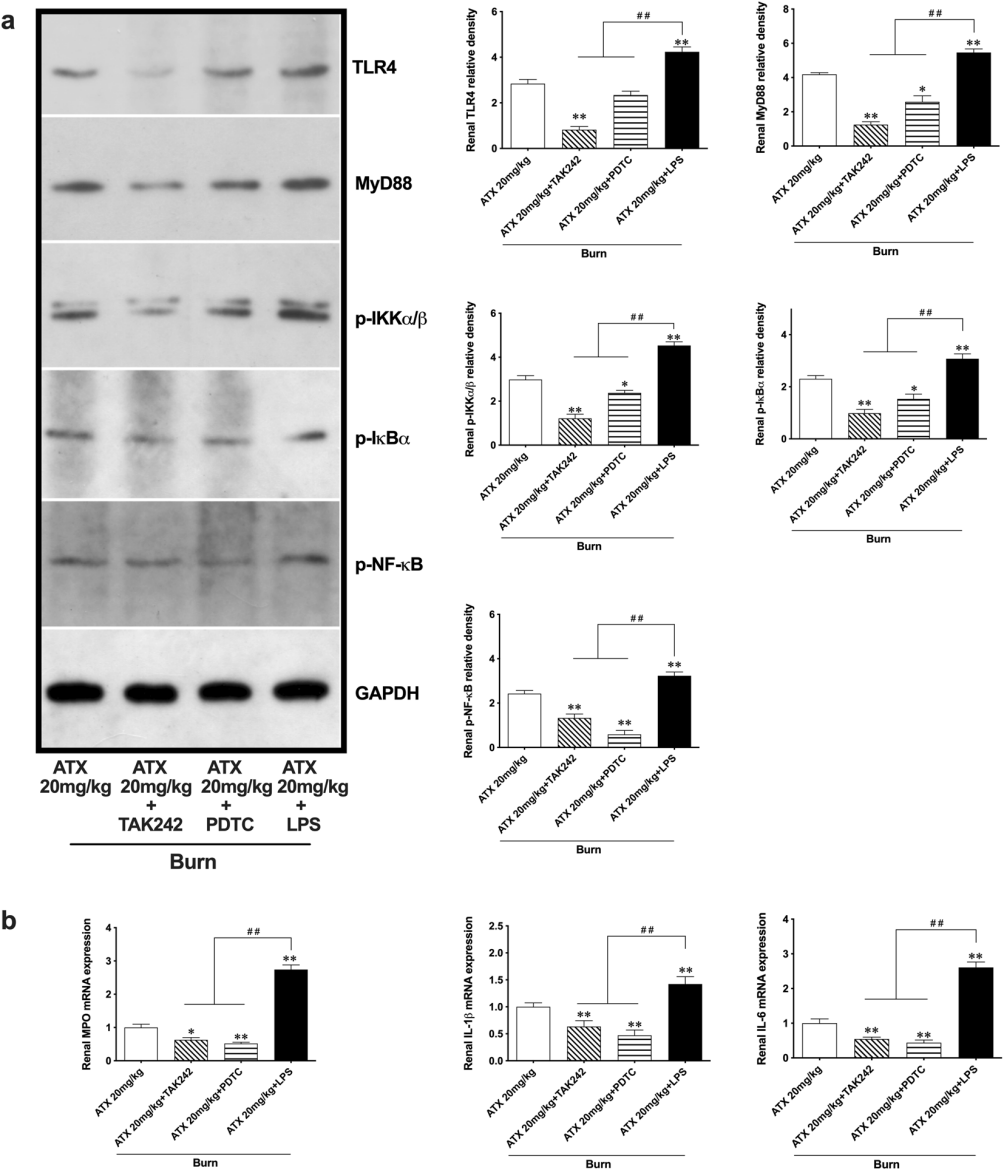
Astaxanthin attenuates burn-induced inflammation through regulating TLR4 /MyD88/NF-κB pathway. (**a**) Representative bands of western blot are shown after introduction of LPS, TAK242 and PDTC. LPS reversed the effect of high-dose ATX on expression of TLR4, MyD88 and downstream phosphorylated activation of IKKα/β, Iκ, BαNF-κB, while both TAK242 and PDTC could help to enhance the effect of ATX; (**b**) The inhibition of ATX on release of selected inflammatory mediators could be abolished by LPS, and enhanceb by using TAK242 or PDTC. n = 6 for each group. The results are expressed as the mean ± SD. ^*^p < 0.05, ^**^p < 0.01, v.s. ATX 20 mg/kg; ^#^*p* < 0.05, ^##^*p* < 0.01.

Combined application of the selective TLR4 activator LPS and TAK242 or PDTC was introduced to determine the anti-inflammatory role of ATX based on the TLR4/MyD88/NF-κB pathway in the progression of burn-induced AKI. The pretreatment with TAK242 assisted high-dose ATX in enhancing the downregulation of TLR4, MyD88, phosphorylated IKKα + β, phosphorylated IκBα, and phosphorylated NF-κB p65 expression, whereas PDTC pretreatment showed a similar but weaker effect on this signaling pathway, with the exception of NF-κB p65 phosphorylation (Fig. 6a). The renal release of selected inflammatory mediators (MPO, IL-1β and IL-6) was also attenuated further with the help of TAK242 and PDTC in the ATX treatment groups (Fig. 6b). On the other hand, compared to the high dose of ATX, simultaneous LPS injection remarkably abolished the downregulation of TLR4 and MyD88 expression and phosphorylation of IKKα + β, IκBα, and NF-κB p65 caused by ATX treatment in the kidneys of burned rats (Fig. 6a). Furthermore, we observed an obvious increase in the mRNA expression of selected inflammatory mediators (MPO, IL-1β and IL-6) in burned rat kidneys treated with the combined application of high-dose ATX and LPS compared to the single application of ATX (Fig. 6b). All these results support an intermediate role of the MyD88-dependent TLR4/NF-κB pathway in mediating the anti-inflammatory effect of ATX.

### ATX dose-relatedly increased the distribution and expression of HO-1 in rat kidneys after burn injury

**Figure 7:**
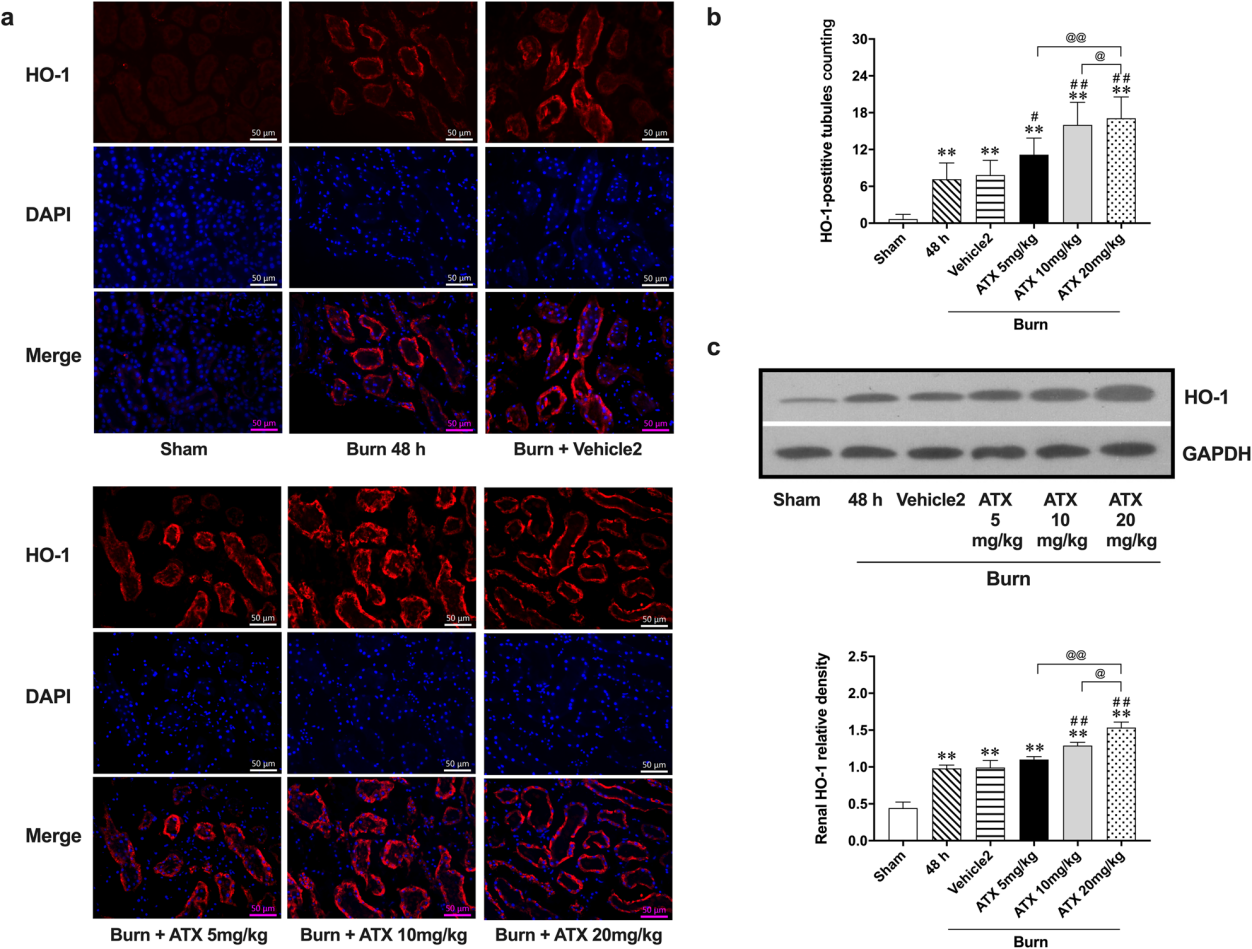
Astaxanthin treatment dose-related increased the distribution and expression of HO-1 in kidneys of burned rats. (**a**) Representative images of Immunofluorescence staining of HO-1 in Kidneys; (**b**) The double-blind counting of HO-1 positively-stained tubules (red-labeled) n = 12 per group; (**c**) Representative bands of HO-1 protein expression, n = 6 per group. . The results are expressed as the mean ± SD. ^*^p < 0.05, ^**^p < 0.01, v.s. sham; ^##^*p* < 0.01, v.s. vehicle2; ^@^*p* < 0.05, ^@@^*p* < 0.01, ^ns^*p* > 0.05.

Based on immunofluorescence staining, we observed increased red-labeled renal tubules 48 h after burn injury, representing HO-1 distribution. According to the count under a microscope, the number of HO-1-positive tubules increased significantly in the 48 h burn group. The administration of medium- and high-dose ATX in burned rats resulted in more red-labeled tubules than observed in the burn group in the selected visual fields, while low-dose ATX showed a weaker ability to further increase HO-1 distribution (Fig. 7a,b). The vehicle of the ATX solution (vehicle 2) showed no effect on burn-induced tubular HO-1 expression (Fig. 7a,b). Furthermore, immunoblot results showed that burn insult induced a significant upregulation of HO-1 expression in the kidneys of burned rats compared to expression in the sham group, while medium- and high-dose ATX administration further increased renal HO-1 expression after burn injury (Fig. 7c). Both vehicle and low-dose ATX showed a slight effect on HO-1 expression upregulation compared to the burn group 48 h after injury (Fig. 7c). Collectively, ATX seemed to further upregulate the renal HO-1 protein level in burned rats in a dose-related manner.

## Discussion

In the present study, we are the first to initiate a systematic investigation of the role of local inflammation and potentially related regulators in the progression of early AKI after burn injury, and to assess the potential protective effect of ATX via its anti-inflammatory effects as a further study following our previous work. Herein, our findings showed the following: (1) burn-induced renal inflammation contributes to the development of early AKI, with a concurrent increase in oxidative stress; (2) the TLR4/NF-κB pathway is involved in regulation of renal inflammation after burn insult and is dependent on MyD88; (3) ATX dose-relatedly attenuates activation of the TLR4/MyD88/NF-κB cascade and the subsequent release of inflammatory mediators; and (4) ATX further upregulates renal expression of HO-1 in a dose-related manner to assist in its regulation of oxidative stress and the TLR4 pathway.

In the early stage after burn injury, the risk of shock accompanied by a reduction in generalized renal blood flow (RBF) causes a series of endothelial and tubular changes, such as endothelial cell swelling and disruption of the endothelial monolayer, as a result of activation of the coagulation system and impaired vascular reactivity, which contributes to early tubular damage post burn injury^1^. In the current study, we observed typical regional changes in the kidneys of a severe burn model over time, including tubular epithelial necrosis and tubular dilation. Moreover, progression of histological damage in the kidneys is accompanied by deterioration of renal function and increases in the classic functional indices sCr and NGAL^22^. A subsequent investigation revealed that burn injury induced a remarkable increase in ROS generation and a decrease in antioxidant enzymes, representing increased oxidative stress 6 h post burn injury, which persisted during the early stage after burn injury, similar to our previous report^8^. The release of inflammatory mediators in kidneys exhibited a time-dependent increase after burn injury and showed a late peak compared with that of oxidative stress, suggesting that it was a result of a continuous ROS burst. After burn injury, leukocytes, endothelial cells and the renal tubular epithelium contribute to generation of several proinflammatory cytokines, such as MPO, IL-6, and IL-1β^8^. Increased release of proinflammatory mediators leads to enhanced vasoconstriction, vessel occlusion and a decreased number of microvessels, which results in edema of the outer medulla, increased tubular injury, and possible tubulointerstitial fibrosis^23^. Previously, we observed clues that suggested a connection between ROS-related oxidative stress and local inflammation in burned rat kidneys, and NF-κB seemed to act as a potential regulator to mediate burn-induced renal inflammation^8^. As a classic signaling cascade, other researchers have exposed the importance of TLR4 and NF-κB in regulating innate and adaptive immune responses to renal ischemic injury^5,23,24^, and the role of the TLR4/NF-κB cascade in ischemic injuries of other organs by regulating immune and subsequent inflammatory responses has been reported^25,26^. Interestingly, we observed that both TAK242 and PDTC seemed to attenuate the histological and functional deterioration of rat kidneys 48 h after burn injury, which provided clues about the potential value of the TLR4/NF-κB cascade in burn-induced AKI. As a specific inhibitor of TLR4, the benefit of TAK242 against histological kidney damage indicates a potential role of TLR4 in burn-induced AKI, while the effect of the NF-κB inhibitor PDTC suggests a pivotal role of NF-κB. Furthermore, administration of either TAK242 or PDTC markedly attenuated the elevated release of four proinflammatory mediators in burn-stimulated kidneys, which suggests a possible effect of the TLR4/NF-κB signaling pathway on regulation of the inflammation related to the development of burn-induced AKI. However, only TAK242 decreased the increased ROS level in kidneys and enhanced the activities of antioxidant enzymes after burn injury, indicating a potential relationship between TLR4 and ROS-mediated oxidative stress.

In terms of the typical TLR4/NF-κB signaling cascade, activated TLR4 can induce downstream phosphorylation of IKKs through a MyD88-dependent or MyD88-independent route and further lead to disintegration of the IKK complex^27,28^. Subsequently, phosphorylation of IκBα favors degradation of IκBα and subsequent dissociation of the NF-κB p65 subunit from the IκBα/NF-κB/Rel complex^27,28^. The dissociated p65 subunit is activated via phosphorylation and then translocates into the nucleus, which ultimately mediates the transcription of multiple genes^27,28^. Several in vivo results from other researchers illustrated that many types of agents display a nephroprotective effect in ischemia–reperfusion-induced AKI by regulating TLR4/NF-κB pathway-mediated inflammation in the kidneys^11,29–31^. In this study, we further investigated the detailed role of the TLR4/NF-κB signaling cascade in burned rats via immunofluorescence staining and western blotting. The time-dependent increase in the distribution and expression of TLR4 and MyD88 suggests that MyD88 is involved in regulating upstream activation of the NF-κB pathway. Subsequently, the renal tissue of rats showed sequentially increased phosphorylation of signals, including IKKα + β, IκBα, and NF-κB p65, along with increased release of proinflammatory cytokines. Combined with the reverse effect of TAK242 and PDTC blockade on renal structure, function and proinflammatory mediators, our results indicate that the MyD88-dependent TLR4/NF-κB signaling pathway is involved in regulation of burn-induced inflammation and related injury in kidneys and might be a pivotal target to prevent or treat burn-induced early AKI.

Astaxanthin (ATX) is a natural and strong antioxidant that can be extracted from multiple crustaceans^32^. ATX is considered to be a safe and hopeful therapeutic agent in several diseases, as well as a safe feed additive and dietary supplement^21,33–35^. Our previous study primarily demonstrated the protective value of ATX in burn-induced early AKI, and the mechanism of action was found to be related to its ability to attenuate oxidative stress and secondary renal cell apoptosis^7^. Inflammation is another crucial result of circulatory or tissue ROS bursts following surgery or severe trauma. ATX has been suggested to have an anti-inflammatory role based on several in vivo and in vitro studies on neurologic injury/dysfunction/disease, dermatitis, lung injury, and diabetes-related disorders, among others^36–42^. In terms of burn injury, we observed a beneficial effect of ATX in preventing deterioration of burn wounds by ameliorating tissue inflammation in wounds^43^. The results of prior studies have suggested that the anti-inflammatory property of ATX might be attributed to regulation of certain inflammation-related signaling molecules, such as NF-κB, JAK/STATS, PI3K/Akt, MAPK, ERK, and MSK, all of which might lead to secondary release of inflammatory cytokines^42,44–48^. In particular, NF-κB has elicited more attention from researchers due to its crucial role in regulating the inflammatory cascade under diverse pathophysiological statuses of multiple organs^27^. Furthermore, based on in vivo and in vitro subarachnoid hemorrhage (SAH) models, Zhang et al. demonstrated that TLR4 plays a crucial role in mediating neuroinflammation caused by insults resulting from SAH, while MyD88 and NF-κB act downstream, and the neuroprotective effect of ATX primarily relies on inhibiting the TLR4 signaling pathway and the subsequent proinflammatory response^36^. In the current study, our observations support the importance of the TLR4/MyD88/NF-κB signaling axis in regulating renal inflammation after burn injury, and ATX exhibited a dose-dependent inhibitory effect on activation of the TLR4 pathway and the secondary increase in the release of inflammatory mediators. Single or combined application of the signal inhibitor TAK242 or PDTC and the classic TLR4 agonist LPS led to enhancement or reversal of the anti-inflammatory effect of high-dose ATX, which further indicates that ATX might ameliorate burn-induced renal inflammation by influencing the TLR4/MyD88/NF-κB signaling pathway.

Similar to apoptosis, inflammation is another event that follows an ROS burst caused by multiple factors. Under trauma or stress, certain inner biological defenses exist to protect against possible organ or tissue injury. The kidney is able to exert adaptive and protective mechanisms to limit harmful effects^5^. As a heat shock protein, HO-1 can be induced rapidly to address both oxidative and cellular stress, presenting powerful antioxidant or anti-inflammatory characteristics^49,50^. Furthermore, previous researchers observed that quick induction of HO-1 expression can be initiated in the kidneys of an AKI model even 3 to 6 h after both ischemia/reperfusion and nephrotoxin insult^13,16^. In the prior and current study, we also detected early upregulation of HO-1 in the kidneys of burned rats, suggesting its potential value in regulating burn-induced AKI. TLR4 is closely related to HO-1 expression, whereas HO-1 can negatively regulate TLR4 by influencing ROS generation or certain signals^51,52^. The antioxidant and anti-inflammatory properties of ATX also rely on activation of HO-1^53,54^. In a streptozotocin-induced diabetic rat model, Yeh et al. found that ATX administration attenuated diabetes-related ocular nerve injury by upregulating HO-1 to decrease oxidative stress and secondary inflammation and that NF-κB acted downstream following HO-1^39^. The beneficial potential of ATX against airway inflammation has also been observed with in vivo and in vitro models and was reported to be closely related to the participation of HO-1^55^. In the present study, burn injury led to a remarkable increase in HO-1 in renal tubules in response to thermal insult, whereas medium- and high-dose ATX further upregulated HO-1. Considering the effect of ATX on ROS production and inflammation in kidneys after burn injury, the obtained data support our proposed role of HO-1 in mediating the ability of ATX to modulate renal inflammation by influencing oxidative stress and TLR4 in the development of early AKI after burn injury.

**Figure 8:**
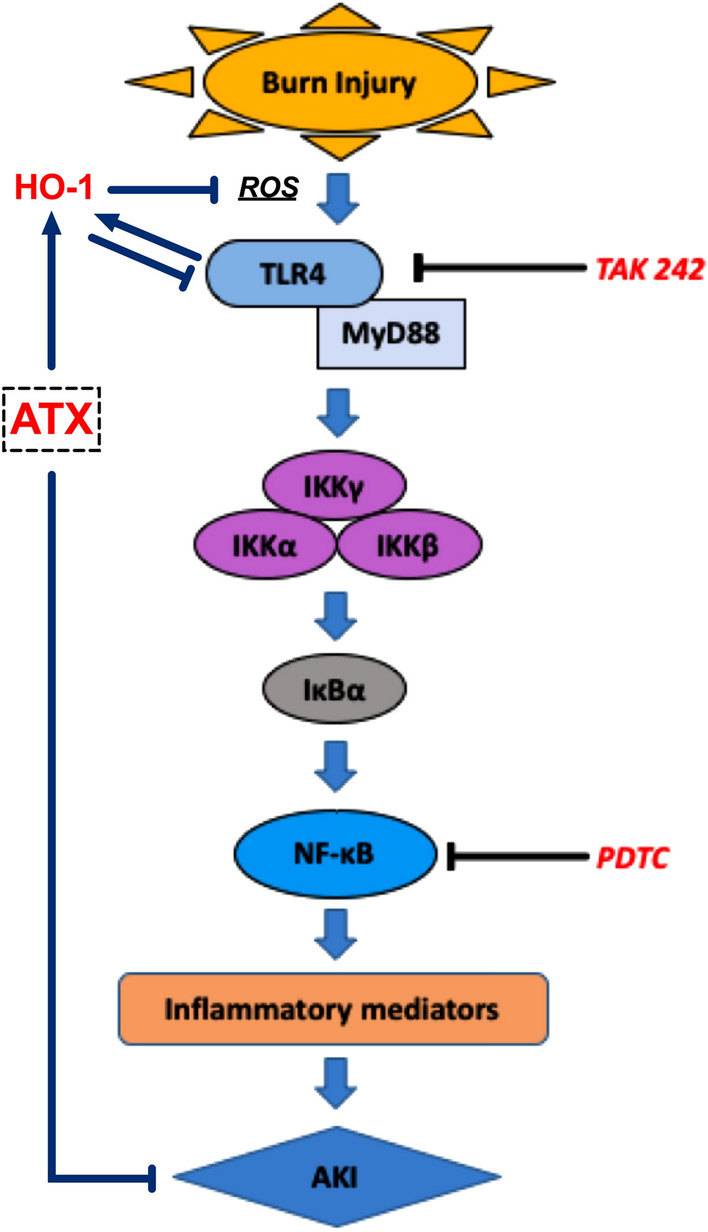
The schematic diagram of this study.

In summary, the current study demonstrated that local inflammation plays an important role in burn-induced early AKI and that the MyD88-dependent TLR4/NF-κB pathway is involved in regulation of renal inflammation (Fig. 8). ATX exhibited anti-inflammation-based protection against early AKI after burn injury, and the beneficial effect can be attributed to its relief of burn-induced oxidative stress and secondary inflammation by influencing the TLR4/MyD88/NF-κB axis. Furthermore, HO-1 acts as a mediator that links the antioxidant and anti-inflammatory effects of ATX. Our study still has some limitations: more work is still required to determine the direct action of ATX on TLR4 or the existence of a potential mediator between ATX and TLR4 signaling; gene-editing technique may help to define the regulation of ATX on signals with less bias; some other signals relating to inflammatory regulation also act downstream of ATX possibly. Nevertheless, our current findings further verify the potential value of ATX as a promising and safe therapeutic agent to improve the outcome of burn patients during the early stage and enrich our knowledge of its mechanism of action.

## Methods

### Experimental animals and severe burn model establishment

The current study was performed according to protocols approved by The Second Affiliated Hospital Zhejiang University School of Medicine Institutional Animal Care and Use Committee (2016-144) and strictly followed the National Institutes of Health Guidelines for the Care and Use of Laboratory Animals. The present study was also carried out in compliance with the ARRIVE guidelines. Adult male 6–7-week Sprague–Dawley (SD) rats weighing approximately 220–250 g were purchased from the Shanghai Slac Laboratory Animal Company (Shanghai, China) and housed with a 12-h light/dark cycle in a filtered-air unit at a constant temperature and humidity. All animals were given free access to food and water. Every animal was housed in one single cage after operation.

The severely burned rat model was established as previously described^7,8^. Specifically, all animals received sodium pentobarbital (50 mg/kg) by intraperitoneal (ip) injection as anesthesia before sham or burn operation. The burn model was established by exposing the rats to a 15 s immersion in 100 °C hot water to generate a large-scale full-thickness dermal burn. The thermally damaged area occupied approximately 40% of the TBSA. The rats in the sham group were treated with 25 °C water on the shaved dorsum after anesthesia. During the operation, the breath and heart rate of burn rats were carefully monitored to ensure that all rats were anesthetized and free of pain before postanesthesia recovery. Liquid resuscitation was preceded by ip injection of lactated Ringer’s solution (LRS) at 4 ml/kg/TBSA immediately and 6 h after the operation. In addition, all rat models were housed in individual cages and administered 0.25 mg/kg buprenorphine via subcutaneous injection immediately and every 12 h postburn for analgesia. A pain and distress scale reported previously was introduced for guiding the analgesia therapy^7^.

### Animal grouping and drug administration

**Figure 9:**
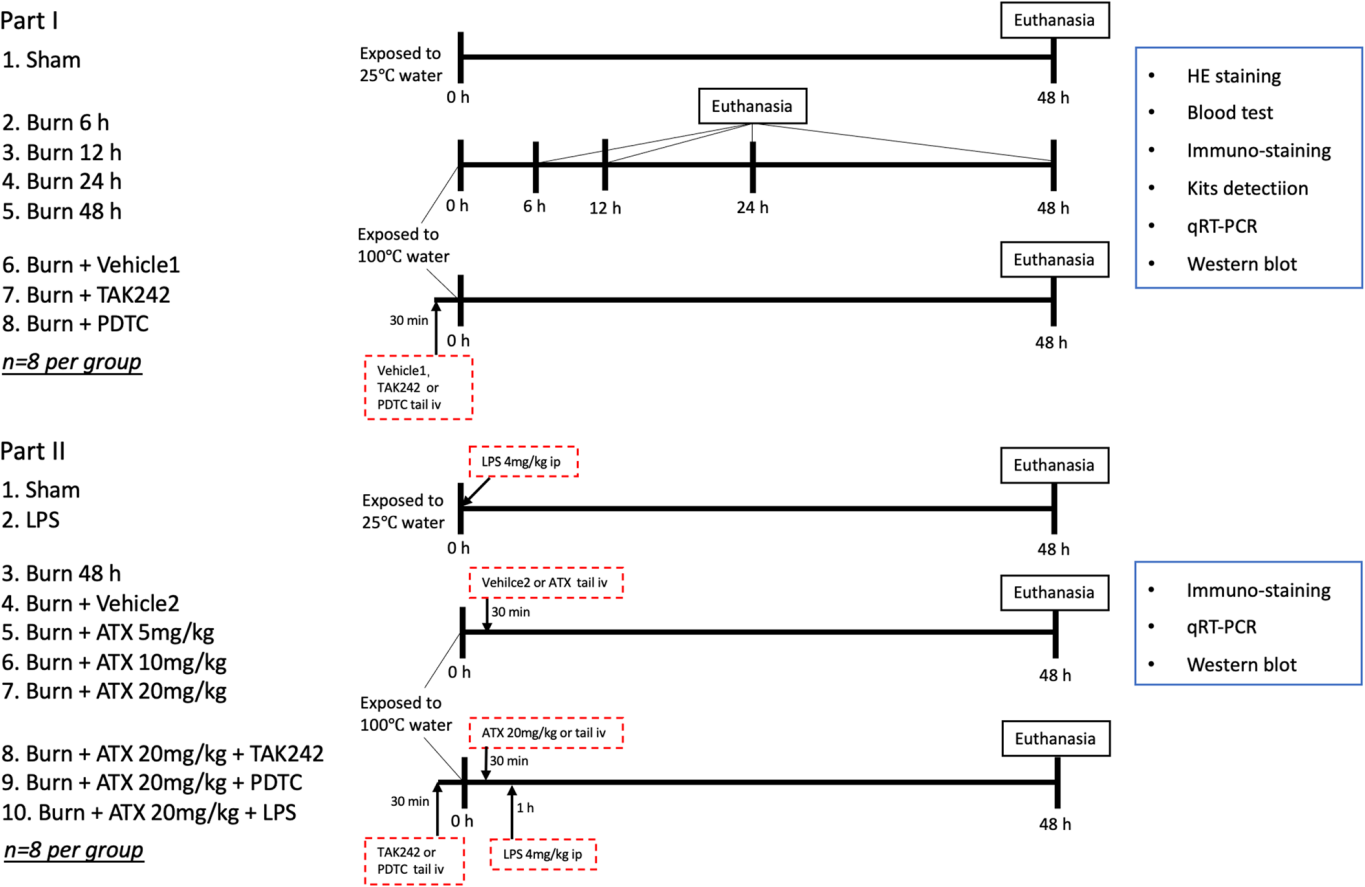
The experimental design and grouping.

The study can be divided into two parts, as shown in Fig. 9. Specifically, in part I, sixty-four SD rats were randomly assigned to eight groups with the help of the random-number table: the sham group, the four burn groups (6 h, 12 h, 24 h, 48 h), the vehicle treatment (burn plus vehicle 1) groups, and the burn plus TAK242 or PDTC groups (n = 8 per group). Both TAK242 (MCE, USA) and PDTC (Beyotime, China) were dissolved in a vehicle solution (vehicle 1, 1% dimethyl sulfoxide in distilled water). Animals in the TAK242 or PDTC group were given TAK242 at a dose of 3 mg/kg or PDTC at a dose of 100 mg/kg by intravenous tail (tail iv) injection 0.5 h preburn^56,57^. Both the sham and burn groups received equal volumes of distilled water, while the burn plus vehicle 1 group was administered an equal volume of vehicle 1 solution. The time-window of observation and sample collection (48 h after sham operation or burn) in controls of sham, TAK242 and PDTC groups was selected according to the results suggesting the most obvious renal injury and renal inflammation found in the 48 h postburn group.

In part II, eighty SD rats were randomly assigned to ten groups with the help of the random-number table: the sham group, the LPS treatment group, the three ATX-treatment burn groups, the burn plus ATX plus TAK242 or PDTC groups and the burn plus ATX plus LPS group (n = 8 per group). ATX (Sigma-Aldrich, St. Louis, MO, USA) solution was prepared in vehicle 2 (polyethylene glycol 400-N,N-dimethylacetamide (PEG400), purchased from Sigma-Aldrich, St. Louis, MO, USA (50:50, v/v)) at concentrations of 5 mg/ml, 10 mg/ml and 20 mg/ml. The rats in the ATX treatment groups received ATX at 5 mg/kg, 10 mg/kg or 20 mg/ml via tail iv injection^7,43^. In the sham group, rats were given equal volumes (1 ml/kg) of distilled water via tail iv injection. The animals in the vehicle 2 groups were given 1 ml/kg PEG400 (50:50, v/v) without any drugs via tail iv injection. All the above treatments were administered 30 min after surgery^7,43^. The LPS control group received 4 mg/kg lipopolysaccharide (LPS) immediately after sham operation via the ip routine^58^. The ATX plus LPS group was given 20 mg/kg ATX 30 min postburn and 4 mg/kg LPS 1 h after burn induction via ip injection^58,59^. TAK242 and PDTC were administered via tail iv injection or intraperitoneally 0.5 h preburn at 3 mg/kg and 100 mg/kg in the selected burn plus ATX groups as mentioned above.

All rats were sacrificed through overdose of sodium pentobarbital. Except for those in the three burn groups (sacrificed at 6 h, 12 h, and 24 h postburn), the animals in the other groups were sacrificed at 48 h post sham operation or burn insult.

### Histological preparation

Following euthanasia, both kidneys were dissected after cardiac perfusion with phosphate-buffered saline (PBS) (pH = 7.2) and were maintained in 4% paraformaldehyde at 4 °C or in a − 80 °C freezer for subsequent assessment^7,8^. Frozen or paraffin-embedded kidney samples were sectioned for further staining.

### Histological examination

Hematoxylin and eosin (HE) staining was performed for histological examination, and the stained slices were observed and imaged under a microscope (Leica, Solms, Germany). Twelve high-magnification files were randomly selected for observation from six slices per group in a blind manner by two pathologists. The tubular damage score was evaluated based on the percentage of injured renal cortical tubules and ranked as 0: normal, 1: less than 10%, 2: 11 to 25%, 3: 26 to 75%, and 4: greater than 75%^7,8^.

### Renal function evaluation

Blood samples were collected to detect the levels of serum creatinine (sCr) by using a clinical chemistry analyzer system and kits as previously reported (Au5800; Beckman Coulter, CA, USA)^7,8^. The levels of serum neutrophil gelatinase-associated lipocalin (NGAL) were determined using a commercial NGAL ELISA kit for RAT (EK0855; Boster, Wuhan, China).

### Immunofluorescence staining

After rewarming, washing and antigen retrieval, the 7-μm-thick frozen slices were separately incubated with anti-TLR4 (1:100; ab22048; Abcam, Cambridge, UK) or anti-HO-1 (1:200; ab13243; Abcam, Cambridge, UK) antibody overnight at 4 °C. After being rinsed with PBS, the sections were incubated with a FITC-labeled goat anti-mouse (1:50; BA1101; Boster, Wuhan, China) or Cy3-labeled goat anti-rabbit (1:50; BA1032; Boster, Wuhan, China) secondary antibody for 2 h at 37 °C in the dark^43^. The sections were rinsed and stained with DAPI (100 ng/ml; Boster, Wuhan, China) for 8 min at room temperature and then mounted with VECTASHIELD mounting medium (H-1000; Vector, CA, USA). All the slices were observed and imaged under a fluorescence microscope (Leica, Solms, Germany). The TLR4-positive (green stained) or HO-1-positive (red stained) tubules were counted under double-blinded conditions. Two independent investigators who were blinded to the group assignments calculated the number of immunostained tubules. At least two visual fields per slide and six slides per group were evaluated by the two investigators.

### Immunohistochemistry (IHC) staining

After deparaffinization, rehydration and antigen retrieval, the sections were incubated with anti-MPO antibody (1:100; ab9535; Abcam, Cambridge, UK), anti-IL-1β antibody (1:100; SC-7884; Santa Cruz Biotechnology, CA, USA), and anti-IL-6 antibody (1:100; SC-1265-R; Santa Cruz, CA, USA) overnight at 4 °C^7,8^. After incubation with goat anti-rabbit secondary antibody (Boster, Wuhan, China), the samples were visualized with a 3,3-diaminobenzidine (DAB) kit (Boster, Wuhan, China). The mounted sections were observed and photographed under a microscope at 200 × magnification (DM2500; Leica, Solms, Germany).

### Oxidative stress assessment

Kidney tissue homogenate was prepared as previously reported^7,8^. ROS generation was detected with a DCFH-DA-based Reactive Oxygen Species Assay Kit (KGT010-1; KeyGEN Biotech, Nanjing, China) and expressed as n-fold compared to that of the sham group. An indirect index of oxidative stress, the malondialdehyde (MDA) level, was measured with a thiobarbituric acid reactive species (TBARS) assay kit (KGT003-1; KeyGEN Biotech, Nanjing, China) and is expressed in nmol/mg protein. SOD activity in the skin tissues of burn wounds was evaluated using a commercial assay kit from KeyGEN Biotech (KGT00150; Nanjing, China). The results are expressed in U/mg protein. Absorbance values were measured using a microplate reader (Model 680 Microplate Reader; BIO-RAD, CA, USA).

### Quantitative real-time polymerase chain reaction (qRT-PCR) analysis

qRT-PCR was performed to determine the mRNA expression levels of myeloperoxidase (MPO), interleukin (IL)-1β, and IL-6. Briefly, total RNA was extracted from frozen kidney tissues with TRIzol Reagent (Invitrogen, Carlsbad, CA, USA) and RNase-Free DNase I (Qiagen, Duesseldorf, Germany)^8^. A SuperScript First-Strand Synthesis System for reverse transcription PCR (Invitrogen, Carlsbad, CA, USA) was introduced to synthesize cDNAs, and RNA and cDNA concentrations, and then, purities were measured via BIO-RAD spectrophotometry (SMARTSPEC Plus, BIO-RAD, CA, USA)^8^. The primers (Table 1) were designed using Primer Premier 6.0 (PREMIER Biosoft, CA, USA) and synthesized by Shanghai Biological Engineering Co., Ltd. (Shanghai, China)^8^. PCR amplifications were conducted with Power SYBR Master Mix (Invitrogen, Carlsbad, CA, USA) in an IQ 5 Real-time PCR system (BIO-RAD, CA, USA)^8^. β-Actin was used as an internal standard to show relative expression levels. Relative quantification of the target gene expression levels was conducted using the 2^−∆∆Ct^ method.

**Table 1:** The oligonucleotide primers used for PCR amplification.

Gene	Genbank accession	Primer sequences (5′ to 3′)	Size (bp)	Annealing (°C)
Rat MPO	NM_001107036.1	GCTACGGGATGGCGATAGGTTT	126	63
		GACACGGTAGTGATGCCAGTGTT		
Rat IL-1β	NM_031512.2	CCTAGGAAACAGCAATGGTCGGGAC	126	63
		GTCAGAGGCAGGGAGGGAAACAC		
Rat IL-6	NM_012589.2	CAATCAGAATTGCCATTGCACAA	169	63
		CGTCTTCGCAAGAGGAAGAGCAGT		
Rat β-actin	EF156276.1	CCAACCGTGAAAAGATGACCCAGAT	97	63
		CCAGAGGCATACAGGGACAACA		

### Western blot analysis

Generally, the lysed protein samples were subjected to SDS-PAGE and transferred onto nitrocellulose membranes via electrophoresis, while aliquots of samples were used to determine the protein concentration of each sample using a bicinchoninic acid (BCA) kit (KGPBCA, KeyGEN Biotech, Nanjing, China)^7,8^. Subsequently, membranes were incubated in blocking buffer for 2 h and incubated overnight at 4 °C with the following primary antibodies: anti-TLR4 (1:200; SC-10741; Santa Cruz, CA, USA), anti-p-IKKα + β (1:500; ab2064; Abcam, Cambridge, UK), anti-IKKα + β (1:1000; ab178870; Abcam, Cambridge, UK), anti-p-IκBα (1:1000; ab12135; Abcam, Cambridge, UK), anti-IκBα (1:500; ab32518; Abcam, Cambridge, UK), anti-p-NF-κB p65 (1:500; #3033; Cell Signaling Technology, Boston, USA), anti-NF-κB p65 (1:1000; ab16502; Abcam, Cambridge, UK), and anti-HO-1 (1:2000; ab13243; Abcam, Cambridge, UK). GAPDH (1:15,000; ab8245; Abcam, Cambridge, UK) was used as a control on the same membranes. Secondary antibodies were applied, and the bands were detected with West Dura Extended Duration Substrate (Pierce, USA) and X-ray film (Kodak, USA). Then relative density of bands (based on gray value) was analyzed using Bandscan 5.0 software (Glyko, USA) via comparison with GAPDH expression^7,8^.

### Statistical analysis

All data are presented as the means ± standard deviation (SD). GraphPad Prism version 7 (San Diego, CA, USA) and SPSS 19 (SPSS, Chicago, IL, USA) were used for statistical analyses. Multiple comparisons were analyzed using one-way analysis of variance (ANOVA) followed by a Bonferroni post hoc test. A value of *p* < 0.05 was regarded as statistically significant.

## Supplementary Information

Supplementary Information 1.

Supplementary Information 2.

## Abbreviations

AKI: Acute kidney injury
IHC: Immunochemistry
IKK: Inhibitor of nuclear factor kappa-B kinase
IκBα: Nuclear factor of kappa light polypeptide gene enhancer in B-cells inhibitor, alpha
IL: Interleukin
IR: Ischemia–reperfusion
IP: Intraperitoneal injection
MyD88: Myeloid differentiation primary response gene 88
NGAL: Neutrophil gelatinaseassociated lipocalin
NF: Nuclear factor
qRT-PCR: Quantitative real time polymerase chain reaction
sCr: Serum creatinine
TLR: Toll like receptor
