# Author Correction: Astaxanthin protects against early acute kidney injury in severely burned rats by inactivating the TLR4/MyD88/NF-κB axis and upregulating heme oxygenase-1

**DOI:** 10.1038/s41598-022-05213-y

**Published:** 2022-01-12

**Authors:** Songxue Guo, Linsen Guo, Quan Fang, Meirong Yu, Liping Zhang, Chuangang You, Xingang Wang, Yong Liu, Chunmao Han

**Affiliations:** 1 Department of Plastic Surgery, The Second Affiliated Hospital Zhejiang University School of Medicine, 1511 Jianghong Road, Hangzhou, 310000 Zhejiang China; 2 Department of Burns, Changzhou No.7 People’s Hospital, 288 East Yanling Road, Changzhou, 213011 Jiangsu China; 3 Clinical Research Center, The Second Affiliated Hospital Zhejiang University School of Medicine, 88 Jiefang Road, Hangzhou, 310009 Zhejiang China; 4 Department of Burns, The Second Affiliated Hospital Zhejiang University School of Medicine, 88 Jiefang Road, Hangzhou, 310009 Zhejiang China; 5 West China Hospital, Sichuan University, 37 Guoxuexiang Street, Chengdu, China

Correction to: *Scientific Reports* 10.1038/s41598-021-86146-w, published online 23 March 2021

The original version of this Article contained an error in the Acknowledgments section.

 “This research was supported by the following grants: National Natural Science Foundation of China (NSFC) No. 81671909 and 8190195, Zhejiang Provincial Natural Science Foundation of China under Grants No. LY18H150004, LY19H150004 and LY20H150010, and Key Research and Development Project of Sichuan Science and Technology Department No. 2018SZ0380.”

now reads:

“This research was supported by the following grants: National Natural Science Foundation of China (NSFC) No. 81671909 and 81901958, Zhejiang Provincial Natural Science Foundation of China under Grants No. LY18H150004, LY19H150004 and LY20H150010, and Key Research and Development Project of Sichuan Science and Technology Department No. 2018SZ0380.”

The original Article has been corrected.

