# Supplementary material for: Astaxanthin protects against early acute kidney injury in severely burned rats by inactivating the TLR4/MyD88/NF-κB axis and upregulating heme oxygenase-1
Source: Sci Rep. 2021 Mar 23;11:6679. doi: 10.1038/s41598-021-86146-w (PMC7988001; doi:10.1038/s41598-021-86146-w)

Astaxanthin protects against early acute kidney injury in severely-burned rats through inactivating TLR4/MyD88/NF-κB axis and upregulating heme oxygenase-1

Songxue Guo[1#], Linsen Guo[2#], Quan Fang[1], Meirong Yu[3], Liping Zhang[4], Chuangang You[4], Xingang Wang[4] and Chunmao Han[4,*]

[1] Department of Plastic Surgery, The Second Affiliated Hospital Zhejiang University School of Medicine, 1511 Jianghong Road, Hangzhou 310000, Zhejiang, China;

[2] Department of Burns, Changzhou No.7 People's Hospital, 288 East Yanling Road, Changzhou 213011, Jiangsu, China;

[3] Clinical Research Center, The Second Affiliated Hospital Zhejiang University School of Medicine, 88 Jiefang Road, Hangzhou 310009, Zhejiang, China;

[4] Department of Burns, The Second Affiliated Hospital Zhejiang University School of Medicine, 88 Jiefang Road, Hangzhou 310009, Zhejiang, China;

[#]The authors contribute equally to this study.

*Authors to whom correspondence should be addressed; E-Mail: zrssk@zju.edu.cn;

Tel/Fax: +86-5710-8776-7187.

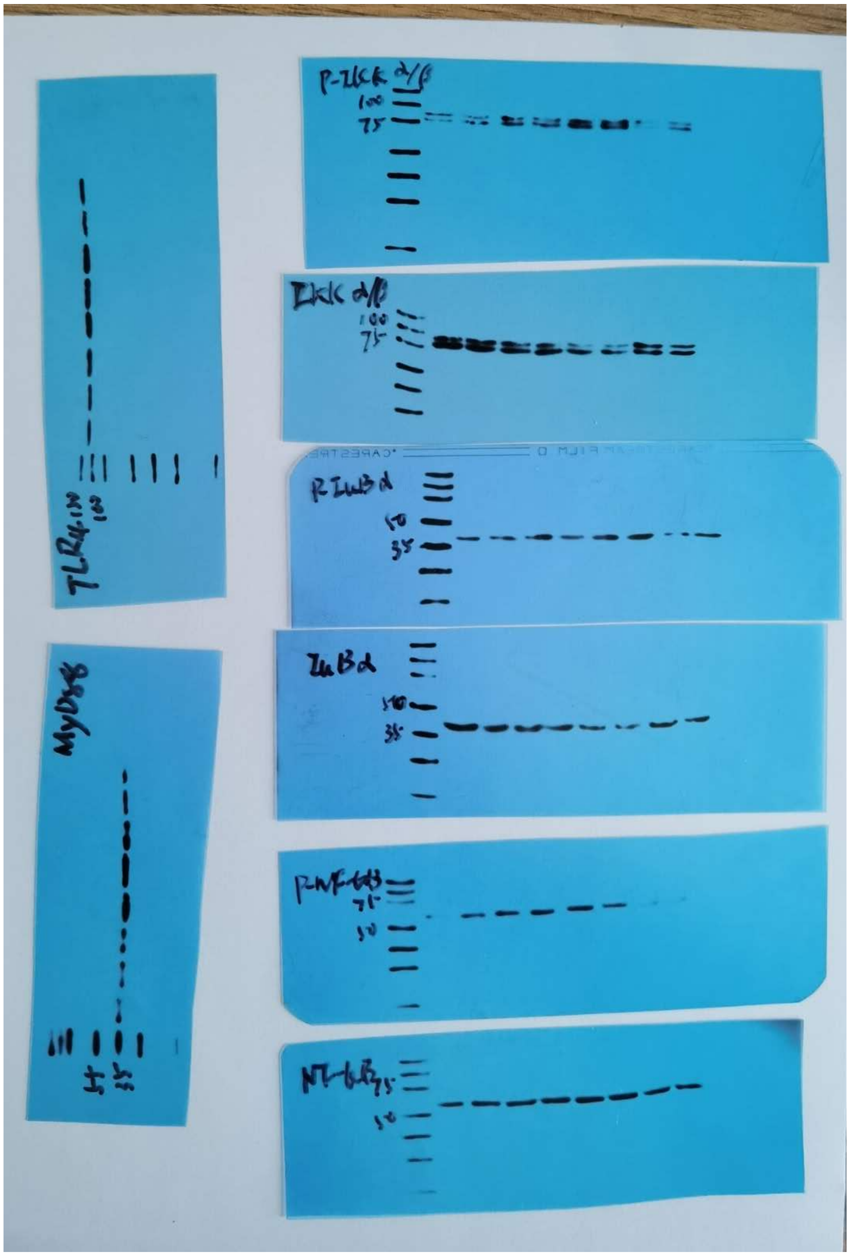
P-IKK α/β
100
75
IKK α/β
100
75
50
35
IκBα
50
35
75
50
75
50
TLR4
MyD88

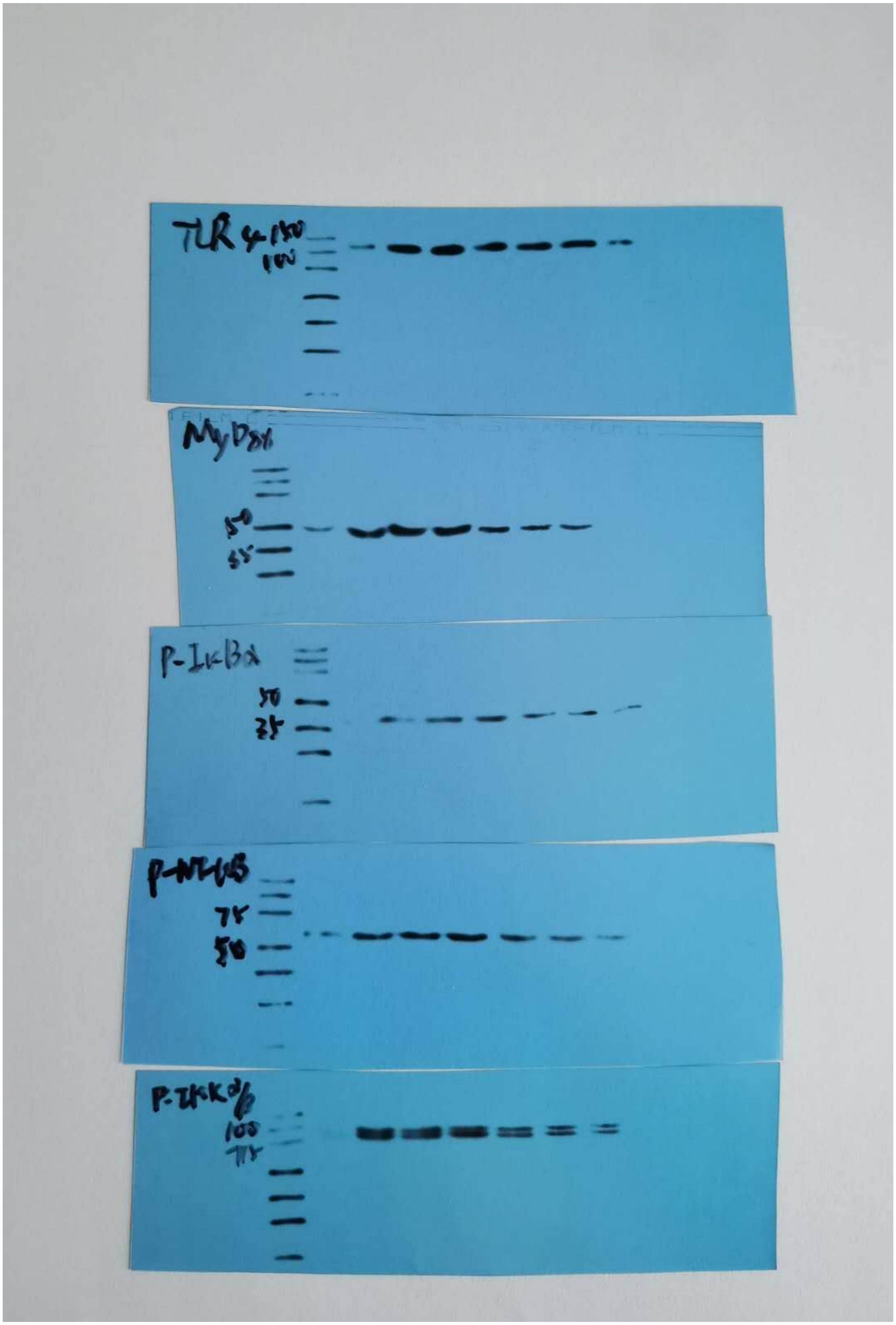
TLR4
150
100
MyD88
50
35
P-IκBα
50
35
P-NFκB
75
50
P-IKKα/β
100
75

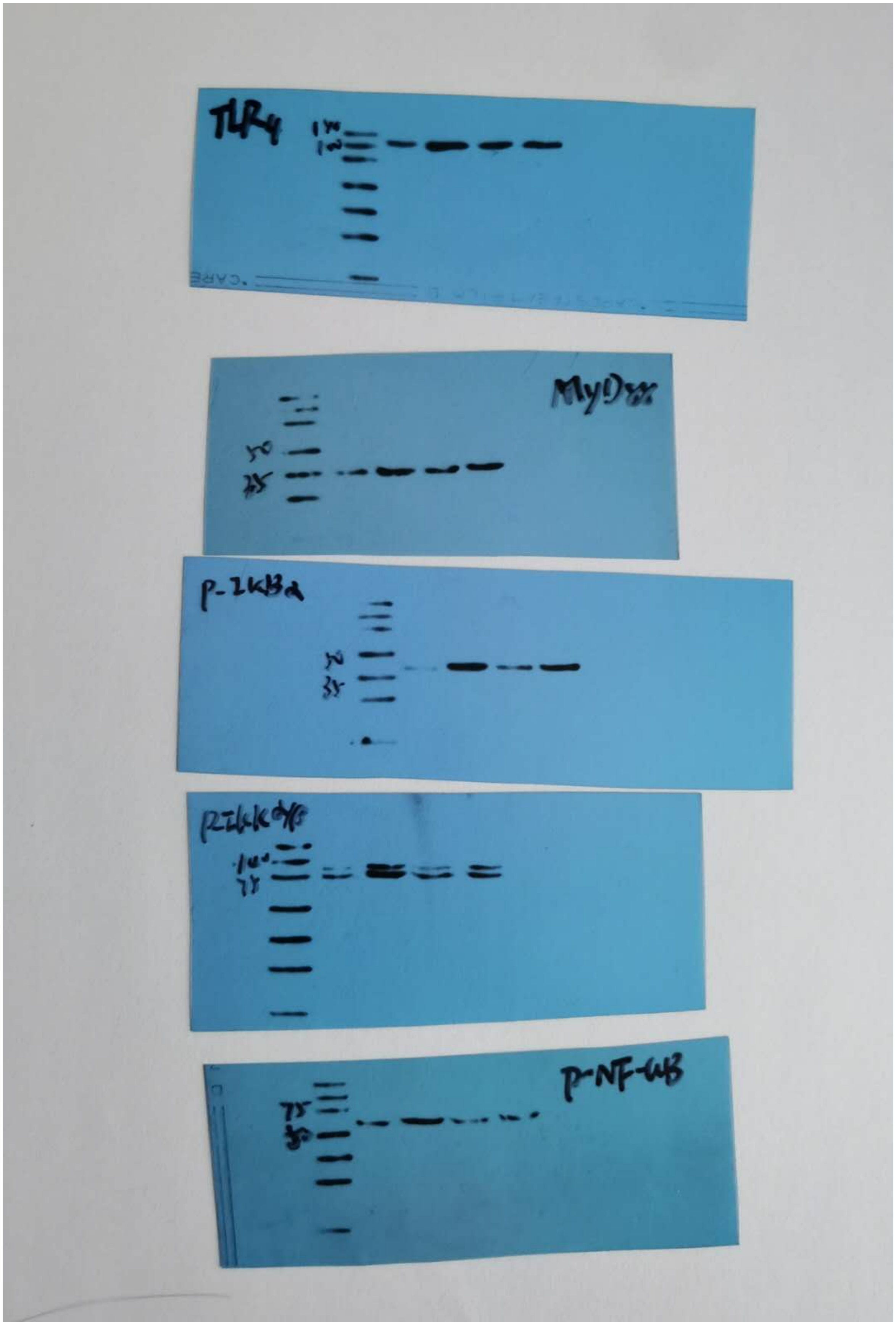

TLR4
MyD88
p-IKBα
p-NF-κB

GAPDH

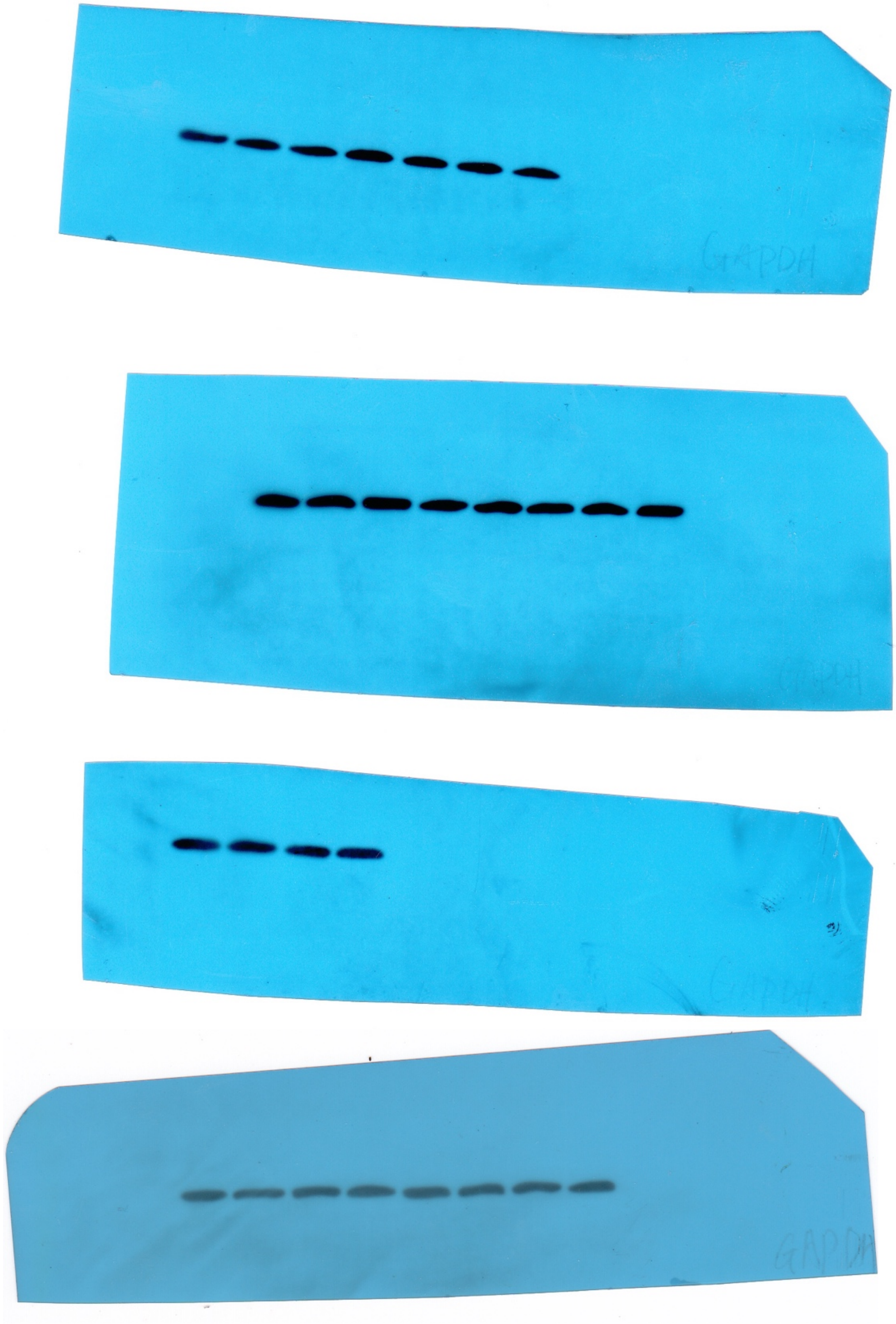

HO-1

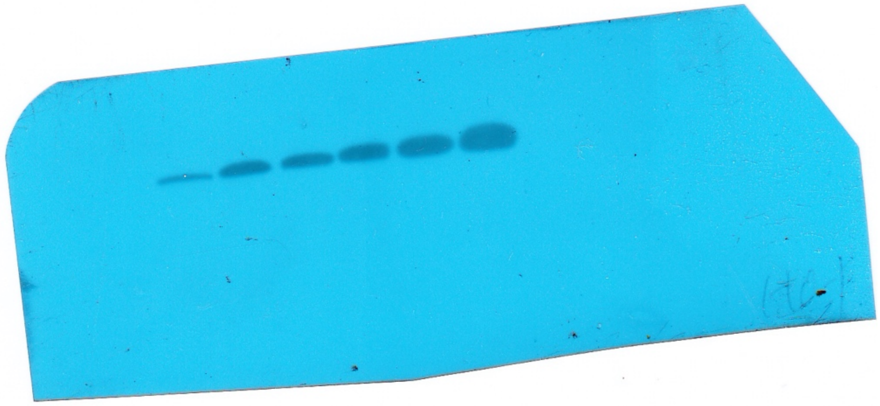

GAPDH

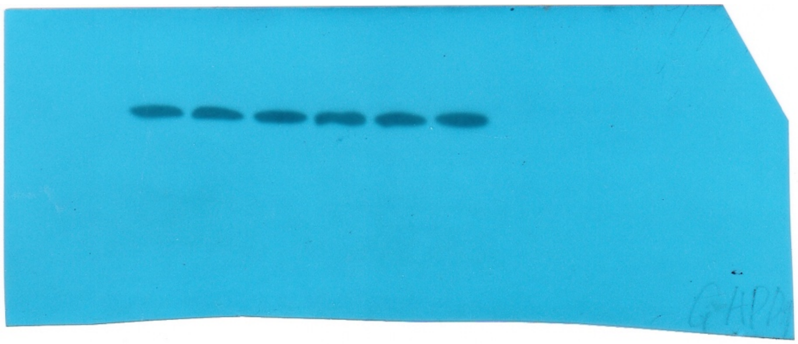

Supplement: Supplementary file 1 — Supplementary Information 1. [file 41598_2021_86146_MOESM1_ESM.pdf]
