# Supplementary material for: Astaxanthin protects against early acute kidney injury in severely burned rats by inactivating the TLR4/MyD88/NF-κB axis and upregulating heme oxygenase-1
Source: Sci Rep. 2021 Mar 23;11:6679. doi: 10.1038/s41598-021-86146-w (PMC7988001; doi:10.1038/s41598-021-86146-w)
Supplement: Supplementary file 2 — Supplementary Information 2. [file 41598_2021_86146_MOESM2_ESM.pdf]

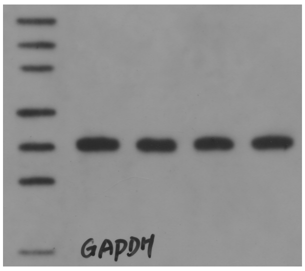
GAPDH

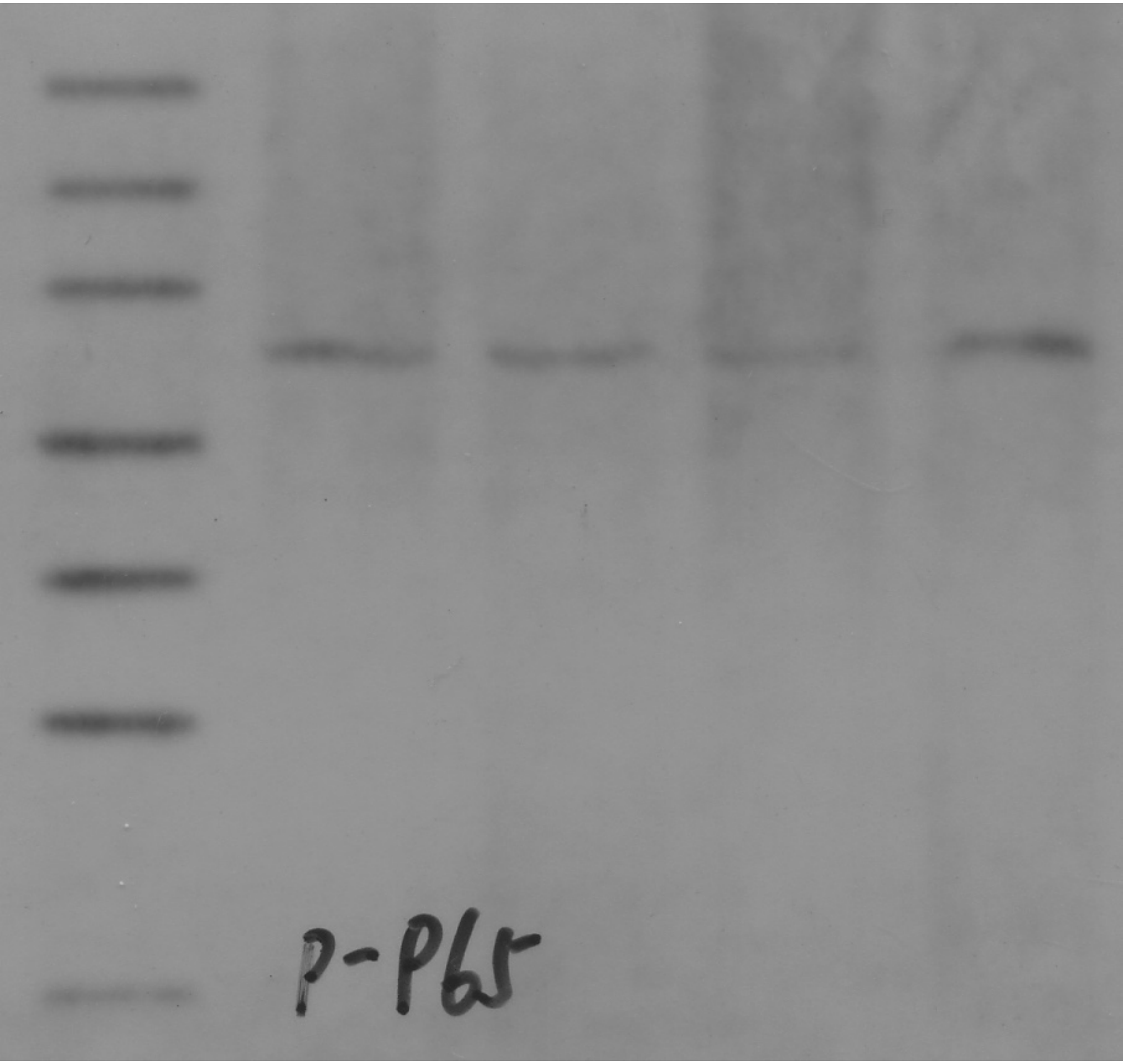
p-P65

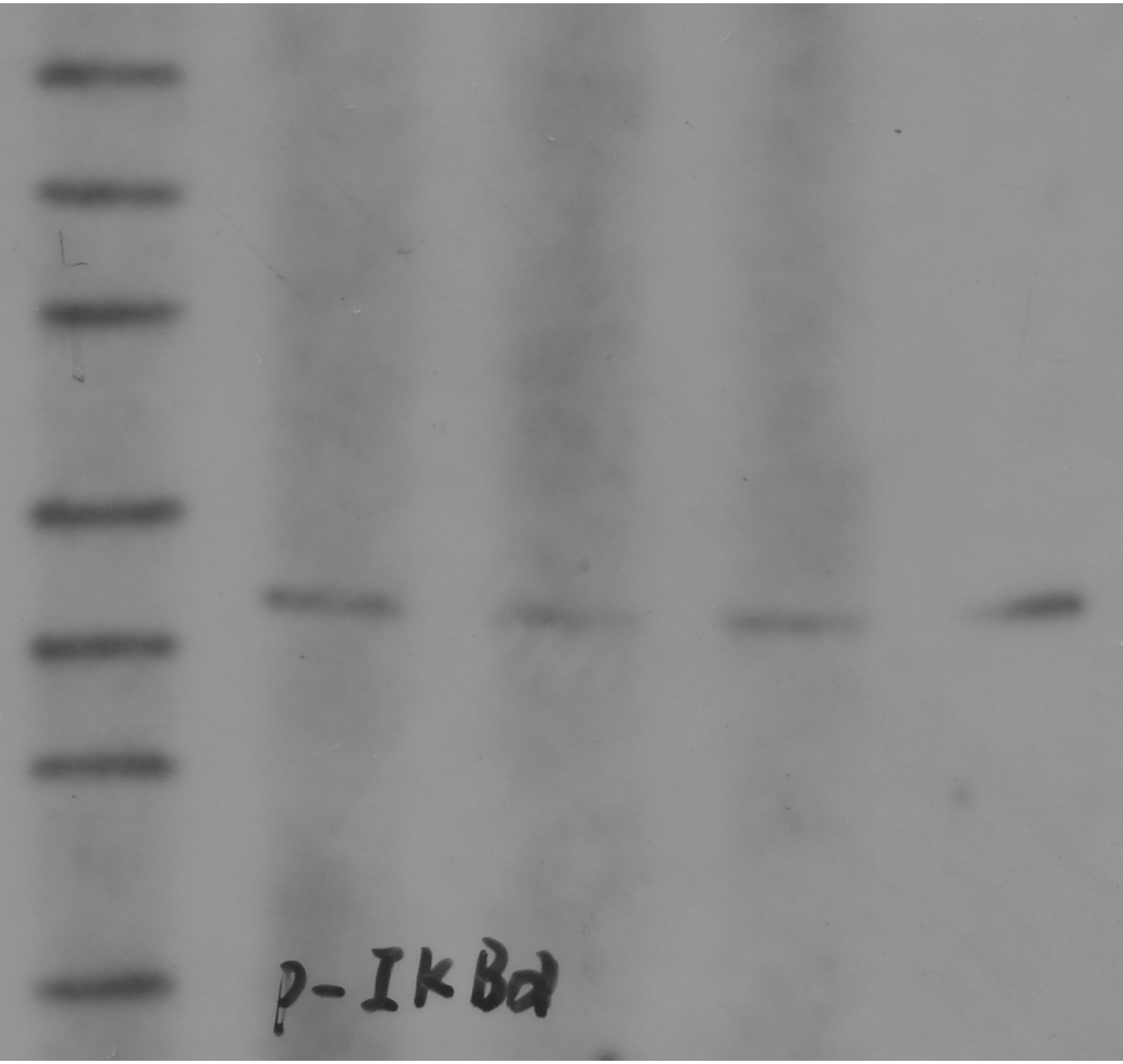
p-IkBa

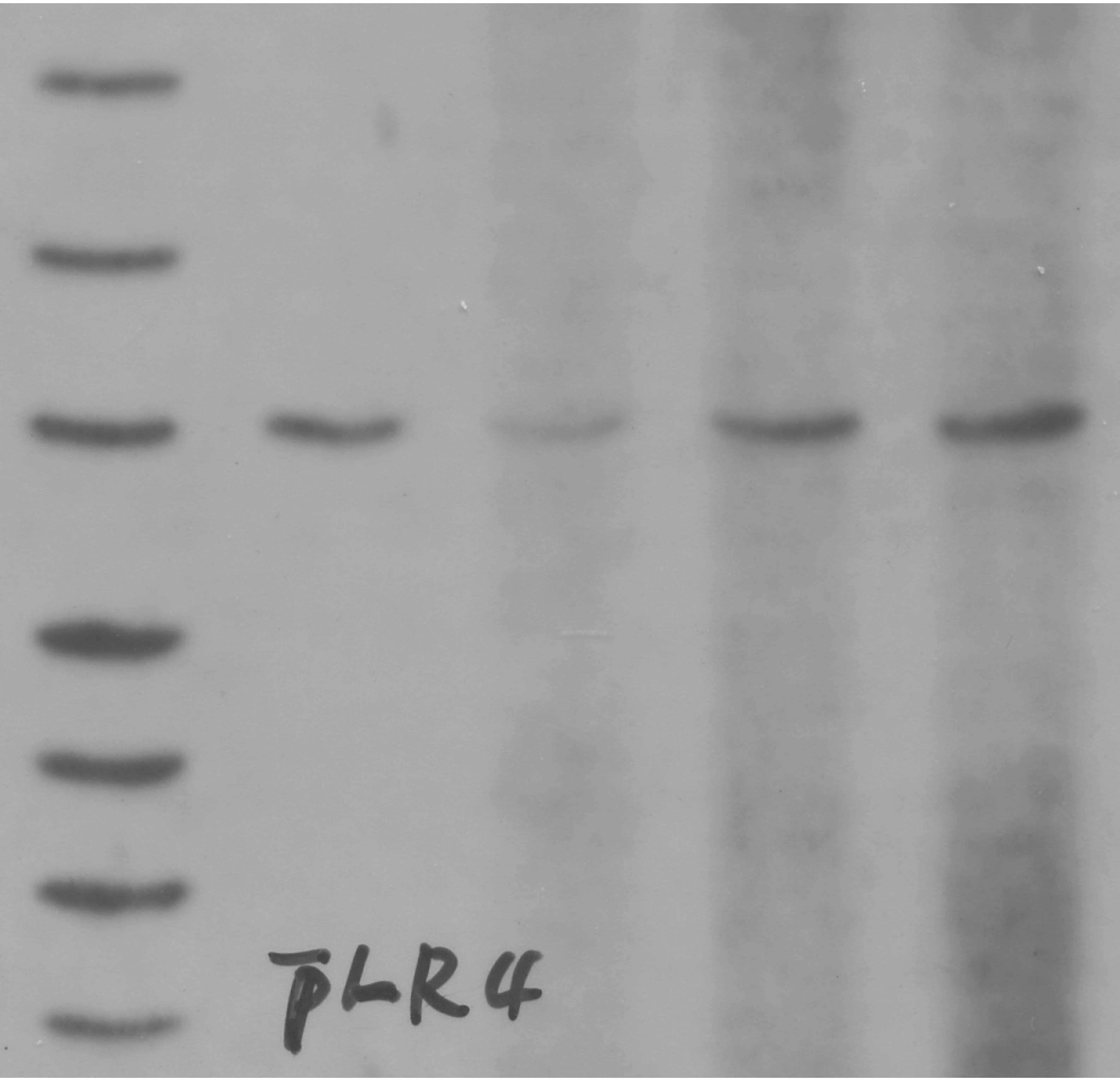
TLR4

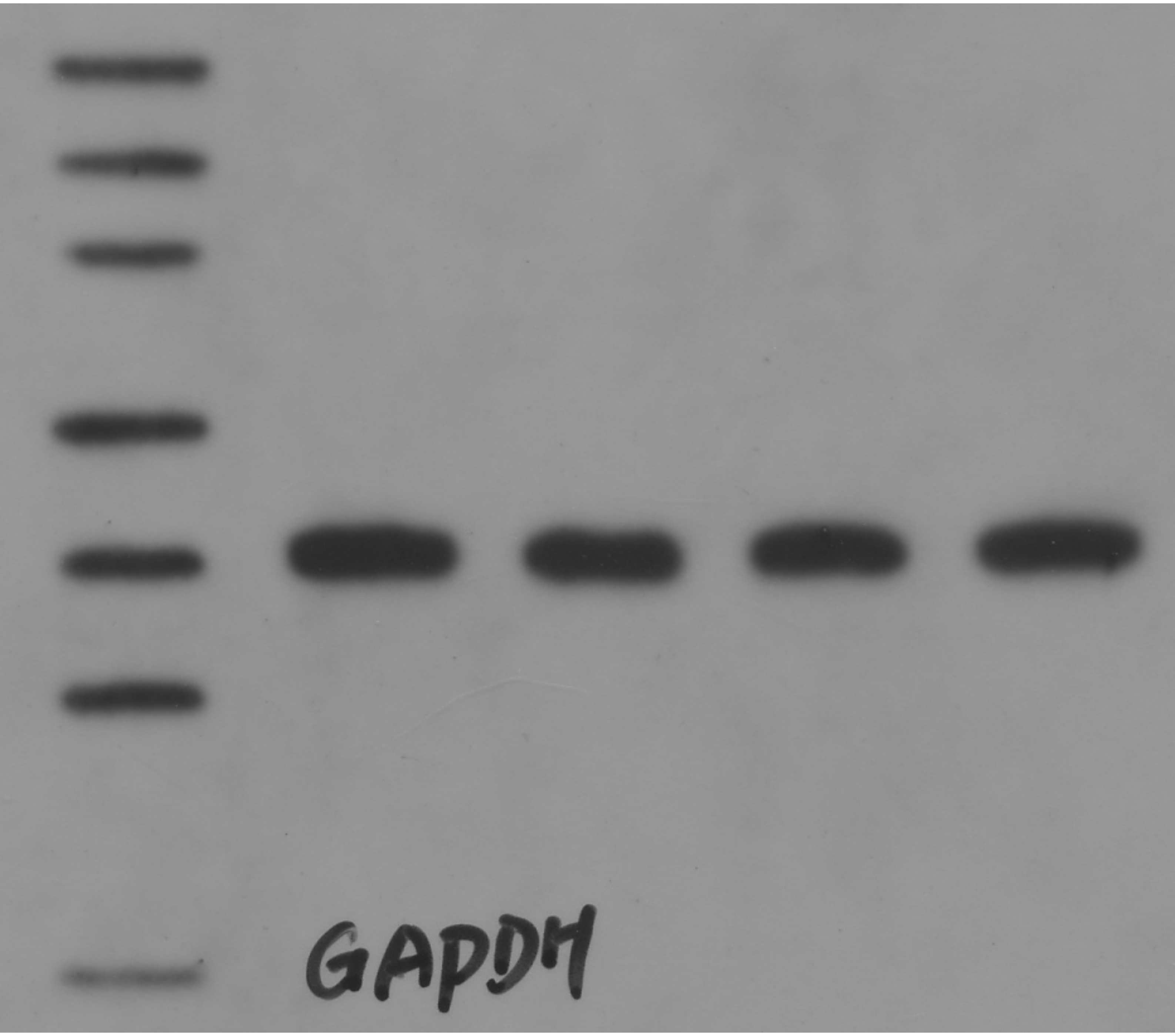
GAPDH

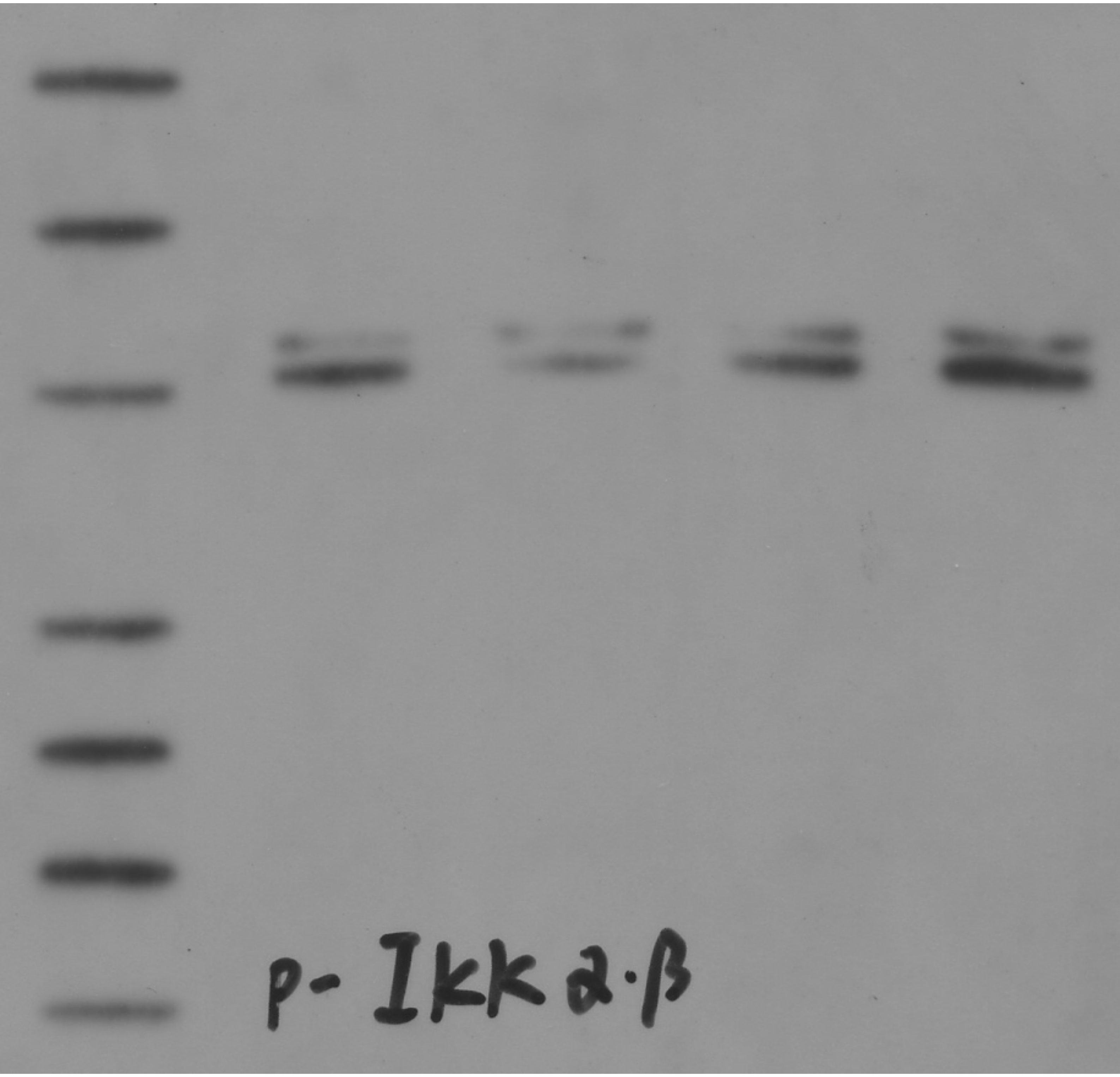
p- IKK α·β
